# Dopamine encodes real-time reward availability and transitions between reward availability states on different timescales

**DOI:** 10.1038/s41467-022-31377-2

**Published:** 2022-07-01

**Authors:** Abigail Kalmbach, Vanessa Winiger, Nuri Jeong, Arun Asok, Charles R. Gallistel, Peter D. Balsam, Eleanor H. Simpson

**Affiliations:** 1 New York State Psychiatric Institute, New York, NY USA; 2 Department of Psychiatry, Columbia University, New York, NY USA; 3 Department of Neuroscience, Columbia University, New York, NY USA; 4 Rutgers Center for Cognitive Science, Rutgers University, Piscataway, NJ USA; 5 Department of Psychology, Barnard College, New York, NY USA; 6 Present Address: Coulter Department of Biomedical Engineering, Georgia Institute of Technology and Emory University, Atlanta, GA USA

**Keywords:** Operant learning, Reward

## Abstract

Optimal behavior requires interpreting environmental cues that indicate when to perform actions. Dopamine is important for learning about reward-predicting events, but its role in adapting to inhibitory cues is unclear. Here we show that when mice can earn rewards in the absence but not presence of an auditory cue, dopamine level in the ventral striatum accurately reflects reward availability in real-time over a sustained period (80 s). In addition, unpredictable transitions between different states of reward availability are accompanied by rapid (~1–2 s) dopamine transients that deflect negatively at the onset and positively at the offset of the cue. This Dopamine encoding of reward availability and transitions between reward availability states is not dependent on reward or activity evoked dopamine release, appears before mice learn the task and is sensitive to motivational state. Our findings are consistent across different techniques including electrochemical recordings and fiber photometry with genetically encoded optical sensors for calcium and dopamine.

## Abstract (web summary)

Dopamine in the ventral striatum conveys a complex reward-associated signal. This study demonstrates how dopamine reflects sustained reward availability, and rapidly signals unexpected changes in availability.

## Introduction

Dopamine (DA) plays key roles in learning, motivation and the regulation of movement. In reinforcement learning paradigms, the magnitude of rapid transient changes in striatal DA that occur at the time of anticipated rewards reflects reward prediction error (RPE), the difference between expected and received rewards^1,2^. In contrast, DA transients evoked by cues that predict reward reflect the expected value of the future reward, as determined by reward probability^3,4^, temporal proximity^5,6^, and magnitude^6–8^. Reward or cue linked phasic dopamine signals can be mimicked using optogenetics to affect learning and modulate conditioned responding^9,10^. However, not all aspects of conditioned behavior are coupled to dopamine responses (refs. ^11,12^) and there is still much to learn about the role of DA in reward-learning. Two important aspects have yet to be fully addressed: the intrinsic negative aspects of task contingencies and the role of dopamine responses in conditioning cues that span durations relevant to naturalistic encounters with reward.

Analysis of dopamine signaling during appetitive reinforcement paradigms has predominantly focused on the positive aspect of task contingencies (S^+^ cues). However, the positive predictive value of any cue also depends on there being negative contingencies, i.e., predictive periods of time during which no outcome is presented^13,14^. In fact, Rescorla suggested 5 decades ago that the strength of an excitatory conditioned response depends on the probability of an outcome being greater in the presence versus absence of a cue^15^. Consequently, excitatory conditioning depends on the animal knowing when outcomes do not occur as well as when they will be presented. Therefore, while dopamine correlates of reward-related behaviors are typically studied by analyzing dopamine responses to positively conditioned cues, an equally important aspect of this learning may involve dopamine responses to cues (deliberate or deduced) that are negatively correlated with reward. To date, the study of dopamine encoding of negative events in reinforcement learning paradigms has been limited to: (1) When an expected reward is not delivered, or is smaller than expected i.e., a negative RPE^10^. (2) When a cue predicts an aversive outcome, e.g., an air puff^16^. (3) When a Pavlovian cue predicts no reward in the presence of a CS^+^ that when presented alone predicts reward, i.e., inhibitory conditioning^17^. However, to our knowledge, there has been no investigation of the relationship between dopamine and cues that inhibit ongoing instrumental behavior and thereby shaping the organization of behavior. This is an important relationship to study considering that deficits in behavioral inhibition are observed in ADHD, addiction, and schizophrenia^18^.

The additional gap we sought to address here arises, in part, from the methods typically employed to study the relationship between DA and behavior. At the behavioral level, reward-learning paradigms typically involve reward-predicting cues that last for only a few seconds. However, conditions associated with reward opportunity in real life are not restricted to such short intervals. At the neurobiological level, electrophysiological or electrochemical methods are ideally suited to provide information about rapid DA changes on the order of seconds. Alternatively, microdialysis typically resolves changes in DA over tens of minutes. Consequently, the information obtained in most studies is restricted to DA fluctuations in one or the other of these methodologically created timescales. This may have resulted in the perception that phasic encoding of reward prediction described above, and the tonic encoding of motivational factors^19,20^ are independent processes. However, a study in which dopamine was monitored over timescales of both seconds and minutes suggests that this is not the case. Apparent changes in the magnitude of phasic DA signals that correlate with RPE may result from changes in baseline DA, from which the amplitude of phasic signals are calculated, but which dynamically changes between trials^21^. Therefore, the functional relationship between DA changes on different timescales may be very important in understanding the dopaminergic regulation of behavior.

In this work, we aimed to resolve both these gaps in the literature, by using a discrimination task in which the unavailability of rewards is signaled for periods longer than a minute. We employed a variety of techniques to measure DA activity and find converging evidence that on separate timescales, DA encodes reward availability states of relatively long duration as well as transitions between such states. To determine if these DA signals play a role in learning, we analyzed the relationship between the appearance of changes in DA release and behavioral adaptation to signaled reward availability. We also investigated how these DA signals were affected by expectations and motivation.

## Results

### Mice learn to inhibit responding during 80-s-long negative discriminative stimuli

**Fig. 1:**
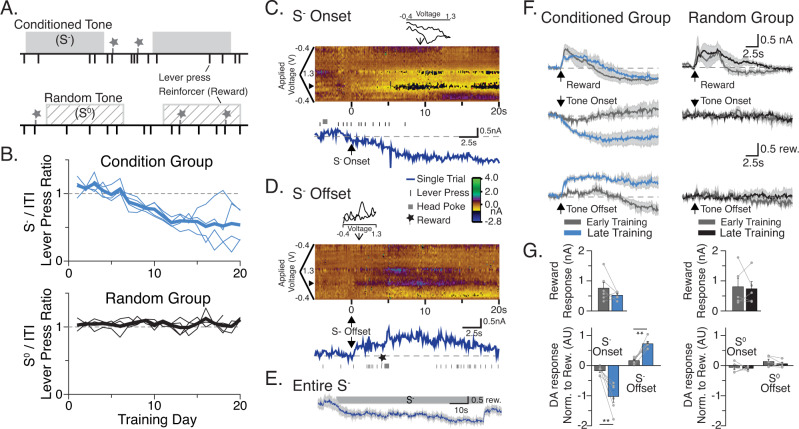
Dopamine encodes reward availability states. **A** Schematic of conditioned inhibition paradigm, S^−^ = conditioned stimulus, S^0^ = Random stimulus. **B** Behavior changes in response to conditioned tone (S^−^; *N* = 4 mice) and random tone (S^0^; *N* = 4 mice). **C** NAc DA response to single S^−^ onset using FSCV. **D** Response to single S− offset. **E** Response to entire S^−^ averaged across 20 presentations in same animal and session as (**C**, **D**). FSCV trace was background subtracted at midpoint of S^−^. **F** Average FSCV responses to dipper (reward) and tone onset early and late in training averaged across animals in Conditioned (S^−^) and Random (S^0^) group (S^−^: *n* = 6 fibers, *N* = 4 mice; S^0^: *n* = 5 fibers, *N* = 4 mice), gray shading is s.e.m. Traces are scaled proportional to 0.5 rew(ard), where 1 reward is the average peak response to reward for each day of recording. **G** Quantification of (**F**): paired two-tailed *t* test: Conditioned group only, DA response to S^−^ Onset Early vs. Late: *t* (5) = 4.544, *p* = 0.006. DA response to S^−^ Offset Early vs. Late: *t* (5) = 6.407, *p* = 0.0014. Error Bars represent s.e.m. **p < 0.01. Source data for (**B**, **G**) are provided as a Source Data file. The raw data used to generate all other figures are deposited in OSF.

We recently demonstrated that the rate and depth of learning about a negative discriminative stimulus (S^−^) is determined by the ratio of the duration of the S^−^ to the intervals between rewards outside the S^−14^. From that study, we selected the training and testing parameters that produced relatively rapid learning and strong behavioral control^14^. We first trained mice in an operant task in which lever pressing is reinforced with milk on average every 20 s (a random interval, RI20, schedule). After mice demonstrated a consistent level of responding, RI20 sessions continued with the addition of presentations of a negative discriminative stimulus, (S^−^). The S^−^ was a fixed-duration 80 s tone during which rewards could not be earned. The S^−^ was randomly presented throughout the session with a variable and unpredictable intertrial interval (ITI) of 40 s (Fig. 1A). Consistent with our previous results, mice trained on an RI20 schedule with an 80 s S^−^ come to respond less during the tone relative to the ITI (Conditioned group, S^−^, Fig. 1B top). In contrast, a separate cohort of mice presented with the same 80-s tone randomly throughout the sessions, without any consistent relation to reward availability continued to respond equally during tone-on (S^0^) and tone-off (ITI) periods (Random group, S^0^, Fig. 1B bottom).

As in our previous study, the average decrease in the ratio of lever presses during the S^−^ relative to the ITI occurs because individual conditioned subjects show a decrease in lever pressing during the S^−^ and/or an increase in lever presses during the ITIs (Supplementary Figs. 1, 2). Together, these data demonstrate that the significance of an 80 s negative discriminative stimulus can be learned. Furthermore, mice learn this task at a rate suitable for us to study neural correlates of performance and acquisition.

### Steady-state dopamine level encodes reward availability in real-time

To determine how striatal dopamine (DA) release is involved in our behavioral discrimination task, we implanted carbon-fiber microelectrodes into the ventral striatum (predominantly the lateral nucleus accumbens core (NAc), Supplementary Fig. 3) and recorded changes in extracellular DA using fast scan cyclic voltammetry (FSCV). In conditioned mice, we observed changes in DA time-locked to S^−^ presentation including a decrease in extracellular DA level that begins at S^−^ onset (Fig. 1C) and a sharp increase in DA levels at S^−^ offset (Fig. 1D). The level of extracellular DA was consistently depressed throughout the entire S^−^ presentation (Fig. 1E and Supplementary Fig. 4) relative to the DA level during the ITI. This pattern of changes in DA was selectively observed in the Conditioned but not Random Group (Fig. 1F, G) suggesting that during our behavioral paradigm, extracellular DA in the ventral striatum persistently encodes the state of reward availability. Because of the relatively slow time resolution of FSCV, we could not determine the significance of the differences in rate at which DA declined and rebounded in this experiment. For this and other reasons, we turned to alternate methods for monitoring dopamine during the acquisition and performance of our behavioral paradigm.

### Mesolimbic dopamine cell activity drives the dopamine encoding of reward availability in ventral striatum

**Fig. 2:**
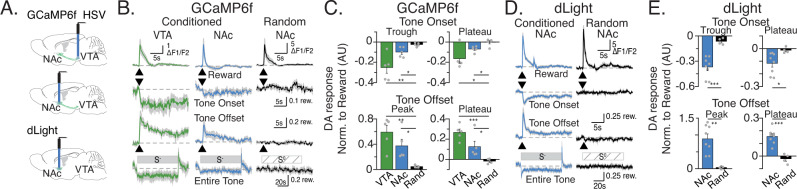
Changes in reward availability are encoded by dopamine transients. **A** Schematic of virus injection and fiber implant locations for photomotery experiments. **B** Average GCaMP6f responses to Reward (dipper), Tone onset, Tone offset and entire tone after training in the Conditioned group in VTA (left, green) the conditioned group in NAc (middle, blue) and Random group in NAc (Right, black). *N* = 5 mice per condition. **C** Quantification of (**B**). S^−^ onset “troughs”: minimal value within 2 s of Tone onset. Tone offset “peaks”: maximal value within 2 s of offset; Plateau DA levels: average of last 60 s of S^−^ for tone onset and 5–7 s after S^−^ offset; trials with rewards within 5 s of offset were excluded. Unpaired two-tailed *t* test results for VTA vs. Random: tone onset trough *t* (8) = 3.97, *p* = 0.0041, tone offset peak *t*(8) = 5.001, *p* = 0.0011, tone onset plateau *t* (8) = 3.249, *p* = 0.0117, tone offset plateau *t* (8) = 8.138, *p* < 0.0001. NAc vs. Random: tone onset trough *t* (8) = 3.199, *p* = 0.0143, tone offset peak *t* (8) = 3.149, *p* = 0.0136, tone onset plateau *t*(8) = 3.299, *p* = 0.0126, tone offset plateau *t* (8) = 2.598, *p* < 0.0317. **D** Average dLight responses (S^−^: *N* = 8 animals; S^0^: *N* = 5 animals). **E** Quantification of responses plotted in d. Unpaired two-tailed *t* test results for NAc vs. Random: tone onset trough *t* (10) = 4.814, *p* = 0.0007, tone offset peak *t* (10) = 4.069, *p* = 0.0023, tone onset plateau *t* (10) = 2.381, *p* = 0.0386, tone offset plateau *t* (10) = 5.459, *p* < 0.0003. Data presented as mean ± s.e.m. b,d, gray shading is s.e.m. Traces are scaled proportional to 0.1 rew(ard) or 0.2 rew(ard), where 1 reward is the average peak response to reward for each day of recording. *p < 0.05; **p < 0.01; ***p < 0.001. Source data for (**C**, **E**) are provided as a Source Data file. The raw data used to generate all other figures are deposited in OSF.

Given recent work suggesting that DA release may be regulated locally within the NAc^22^, we sought to further probe if the encoding of reward state by extracellular NAc DA is also reflected in the activity of the mesoaccumbal DA cells projecting to the NAc. Thus, we injected a retrograde herpes-simplex virus encoding a cre-inducible fluorescent calcium indicator, GCaMP6f, into the NAc of DAT-ires-cre mice using the same coordinates used for FSCV recordings. In the same surgery, we implanted optical fibers above the cell bodies in the VTA or above the axonal projections in NAc to image calcium transients using fiber photometry (Fig. 2A top-middle, Supplementary Fig. 3^23^);. We found steady-state encoding of the S^−^ and ITI periods in both DA cell bodies and DA axons. Similar to what we observed with FSCV (Fig. 1E, F), GCaMP6f level during the S^−^ was significantly lower than during the ITI. Specifically, we compared the S^−^ onset plateau (calculated as the average of the last 60 s of the S^−^) with the S^−^ offset plateau (calculated as the average of seconds 5–7 following S^−^ offset, to avoid contamination with potential fast transient offset responses). To avoid contamination with reward-evoked DA responses, any trials in which a reward was delivered within the first 7 s after S^−^ offset were excluded from the S^−^ offset plateau average (Fig. 2B; for individual animal’s DA response see Supplementary Figs. 5, 6). Because we used retrograde targeting, our GCaMP6f signals in VTA came exclusively from DA cells that project to the area of the ventral striatum in which we injected the virus, the same location we used for NAc recordings. Therefore, the similarity of our recordings in the VTA and NAc (Fig. 2B, C) suggests that in this paradigm DA encoding of reward availability does not depend on local regulation of axonal release within the ventral Striatum.

Employing both FSCV and fiber photometry during the same behavioral procedure identified additional information. While the general pattern of changes in DA and GCaMP6f were very similar, some differences, potentially due to differences in spatial or temporal resolution or limits of detection, were observed. Compared to DA levels measured with FSCV, fiber photometric recordings of GCaMP6f demonstrated faster transitions at S^−^ onset and offset towards the steady-state levels of activity that reflect reward availability. Furthermore, with GCaMP6f in either the VTA or NAc we observed an additional component of the S^−^ offset response. At the offset of the tone, there was a sharp spike in GCaMP6f signal which decayed into the higher plateau levels that were associated with reward availability (Fig. 2B, C).

### The Biosensor dLight reveals additional components to dopamine encoding of changes in reward availability

To further expand on our understanding of the dynamics of the DA response, we applied a third technique to measure changes in dopamine during the same behavioral task. The genetically encoded dopamine sensor (dLight) reports changes in extracellular DA with broader spatial, and finer temporal resolution than FSCV^24^. We injected an adeno-associated virus encoding dLight into the ventral striatum and implanted a fiber optic probe above the injection site (Fig. 2A bottom; Supplementary Fig. 3). Consistent with FSCV and GCaMP6f results, dLight measures of DA release were consistently lower during the 80 s S^−^ presentations, compared to the ITIs. We also observed a fast, transient increase in DA release at S^−^ offset, similar to GCaMP6f (Fig. 2D, E for summary, Supplementary Fig. 7 for individual subjects). However, dLight transients at S^−^ offset were faster than GCaMP6f transients (Supplementary Fig. 8). In addition to these signature changes, uniquely with dLight, we observed a rapid decrease in DA at S^−^ Onset that was transiently deeper than the sustained DA decrease maintained throughout the duration of the S^−^ presentation (Fig. 2D, E for summary, Supplementary Fig. 7 for individual subjects, Supplementary Fig. 8 for onset rise time). Thus, dLight revealed that seconds long DA transients symmetrically encode the transitions into and out of different reward availability states.

### Positive and negative dopamine changes develop symmetrically with training

**Fig. 3:**
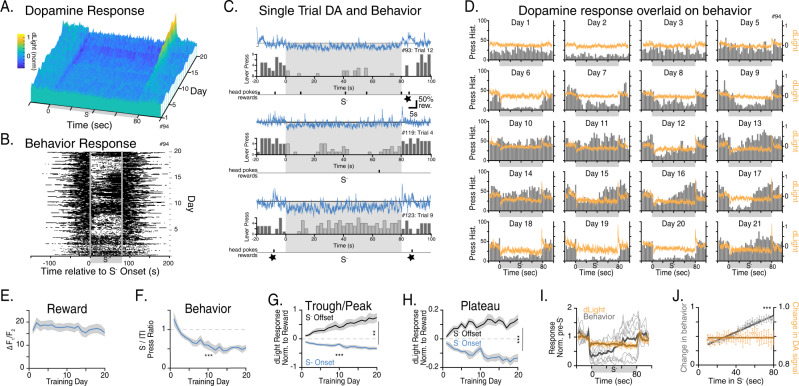
Dopamine dynamics evolve with training but do not predict behavioral dynamics. **A** DA encoding of S^−^ develops gradually across training days as measured with dLight in a single subject. Each day is the average of 20 trials. **B** Behavior changes in response to S^−^. Each row represents a trial. Each circle represents one lever press. **C** Representative dLight traces and behavior (lever presses in 2.5 s Bins) for 3 single trials from the same subject and session. **D** Overlay of dLight (average of 20 trials from a single session) and behavior (histogram counts of lever presses in 2.5 s bins accumulated across 20 trials) for each day of recording. **E** Average dLight response across subjects to reward (dipper up) across training, One-way ANOVA *F* (4.256, 29.79) = 1.556, *P* = 0.2093. **F** Development of behavioral response to S^−^, *F* (3.666, 25.66) = 8.988, *P* = 0.0002. **G** Development of DA transients (troughs and Peaks), two-way ANOVA: Session *F* (19, 133) = 5.957, *P* < 0.0001, tone condition *F* (1, 7) = 27.31, *P* = 0.0012. **H** Development of DA plateaus after S^−^onset (during tone) and S^−^ offset (during ITI), two-way ANOVA: tone condition *F* (1, 7) = 58.17, *P* = 0.0001. **I** Comparison of behavior and dLight in 2.5 s bins for the last 6 days of training for single subject. **J** Within tone dynamics of behavior vs. dLight during the last 70 s in tone averaged across subjects. Best fit of slope compared to zero (Extra Sum of Squares): change in dLight, *F* (1, 230) = 0.2235, *p* = 0.6368, change in behavior, *F* (1, 230) = 225.5, *p* < 0.0001. **A**–**D**, **I** Same subject, Day 4 was excluded due to poor quality recording. **E**–**H**, **I** *N* = 8 mice, gray shading or error bars represent s.e.m. Missing days due to poor recordings were filled in by linear interpolation. **p < 0.01; ***p < 0.001. Source data for (**E**–**J**) are provided as a Source Data file. The raw data used to generate all other figures are deposited in OSF.

To determine how behavior and the multiple components of DA encoding emerge with learning, we examined the trajectory of dLight signals recorded in the ventral striatum daily for a minimum of 20 sessions (Fig. 3A–D). Because rewards were earned during the ITI on an RI20 schedule with an exponential distribution, there are no clear predictors of when reward will be delivered during the ITI. This lack of predictability is likely the reason that dLight responses to reward did not diminish in magnitude as they would for a predictable reward, but instead remained stable across sessions (Fig. 3E). We used the stable reward responses to normalize DA responses to S^−^ each day across training as behavioral conditioning emerged (Fig. 3F). The amplitude of both S^−^ onset troughs (DA minimum value within 2 s of S^−^ onset) and S^−^ offset peaks (DA maximum value within 2 s of S^−^ offset) increase over the entire course of the 20 training sessions (Fig. 3A, G; Supplementary Fig. 9). Plateau values evolved differently with training. The DA plateau value associated with the S^−^ period deepened for approximately the first 10 days before reaching a consistent level. This was mirrored by the DA plateau value associated with the ITI period which increased for approximately the first 10 days before leveling off (Fig. 3H). The symmetry of the tone on and off plateaus is evidenced by a two-way RM ANOVA of the absolute (unsigned) DA plateau values. There is only an effect of session: *F* (19, 133) = 5.484, *P* < 0.0001, no effect of tone condition: *F* (1, 7) = 0.9586, *P* = 0.3602, or a session × tone interaction: *F* (19, 133) = 0.9625, *P* = 0.5088. This symmetrical development suggests that DA release toggles between a high and a low level depending on which of the two possible reward availability states the animal is currently experiencing.

### The dopamine encoding of reward availability does not reflect changes in behavioral output

The decrease in the ratio of S^−^/ITI lever pressing demonstrates an understanding of the relevance of the presence of S^−^. Another expression of that understanding is the temporal dynamics of responding within the S^−^ presentation. After some training, all mice demonstrate a pause in responding early in the S^−^ and resume lever pressing at various times within the S^−^ despite never earning a reward during the tone. Therefore, despite much variability in the pattern of lever pressing on individual trials (see examples in Fig. 3C), when all the presses in each trial are summed across a session, there sometimes appears to be a ramp-like increase in lever pressing during the S^−^, even though animals do not typically show a ramping up of lever pressing within a single S^−^ presentation (for individual subject example see 3B). Aligning dLight signals with lever presses revealed that DA and behavior are uncoupled at the level of single trials, single sessions and also over the course of acquisition. Figure 3C includes 3 representative trials in which a mouse makes very few presses during the S− (top), presses consistently throughout the S− at a similar rate as in the ITI (middle), or makes two distinct bursts of pressing during the S−. Despite these dynamic differences in behavior, the dLight signals show the same pattern in each case: an abrupt dip in DA aligned to S− onset, a consistent trough in DA during the S− that is below the DA level during the ITI and a distinct DA peak at the S− offset.

Overlaying S^−^ dLight and behavior data from a single subject for each session (Fig. 3D) also demonstrates that lever pressing and DA are uncoupled. During many of the sessions, the summed presses for all trials varies across the S^−^ (e.g., in sessions 14, 15, 16 the number of presses increases) but DA remains flat during the S^−^. Also, the pattern of pressing within the S^−^ varies across session (low, high, increasing, etc) while the depression in DA during the S^−^ is the same across sessions, regardless of the press pattern. Therefore, variability in pressing within and across sessions is not mirrored by variability in the DA level during S^−^ (Fig. 3I). DA is at a consistent low plateau for the last 70 s of the S^−^ presentation (slope average = 0), while in some sessions mice display behavioral anticipation of the S^−^ offset with an increase in the probability of resuming lever pressing as the offset of S^−^ approaches, resulting in a positive slope (Fig. 3J). Taken together, these findings suggest that the steady-state DA encoding of reward availability (S^−^ vs. ITI) is uncoupled from the rate of responding.

### The dopamine encoding of reward availability does not depend on activity or reward-related fluctuations in dopamine

We observed a clear uncoupling of DA encoding of reward availability and behavior. To rule out the possibility that this independence was due to averaging of dopamine fluctuations associated with activity and resultant reward presentations, we performed further analysis at the level of individual trials and also individual actions.

**Fig. 4:**
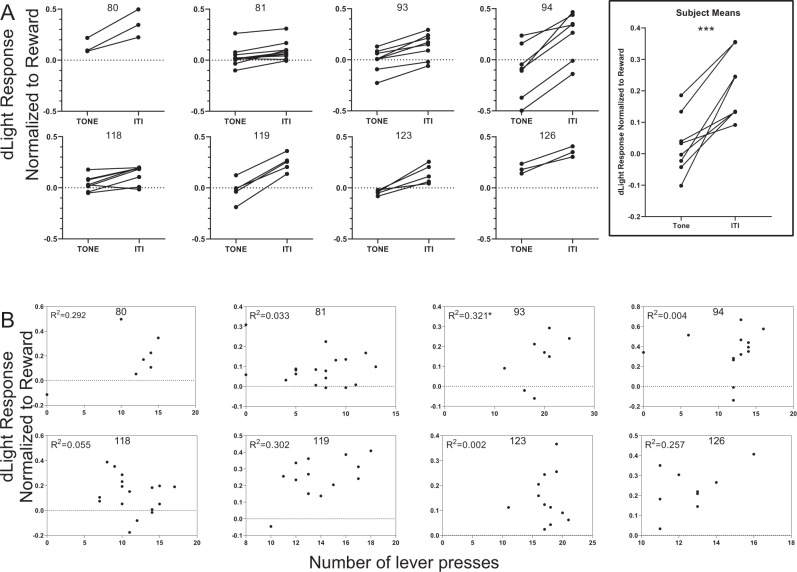
Dopamine encodes reward availability state independent of reward and activity. **A** dLight responses in the last 10 s of S^−^ tone presentations are consistently lower than dLight response in a 10 s period without rewards in the following ITI. Each single subject plot includes the 3–10 trials in which no reward was earned in the first 20 s of the ITI during the last session with dLight recording (session 20). The right most plot depicts the mean S^−^ tone and ITI dLight values for each subject. Paired 2-tailed *t* test: *t* (7) = 5.546, *p* = 0.0009. **B** Each single subject XY plot depicts the dLight response and the number of lever presses made in the same 10 s ITI period depicted in 4a for the last 2 sessions with dLight recordings (sessions 18 or 19 and 20, all post-acquisition). Pearson correlation coefficient, (two tailed) identified a relationship between dLight and lever presses for only one subject, 93: number of pairs:16, *r* = 0.5665, R squared = 0.3209, *p* = 0.0221. *N* = 8 animals. *p < 0.05; ***p < 0.001. Source data for (**A**, **B**) are provided as a Source Data file.

To exclude the possibility that reward-evoked dopamine was responsible for the higher level of dLight signal during the ITI relative to the S^−^ period, we compared the average level of dopamine in the last 10 s of an S^−^ presentation to the average level of dopamine in the 10 to 20th second of the adjacent ITI for trials in which no rewards during the first 20 s of the ITI period. We excluded the first 10 s of the ITI to exclude the large DA transient that is a signature of tone offset, the transients between reward states. Despite the absence of rewards, the DA level during the ITI was consistently elevated compared to the DA level during the preceding S^−^. Figure 4A depicts individual trials from the 20th session for each subject and a comparison of the average values for each subject.

To exclude the possibility that movement related fluctuations in DA are responsible for the higher level of DA during the ITI relative to the S^−^ period, we performed correlation analyses between dLight responses and number of lever presses within individual trials for each subject (Fig. 4B). We computed Pearson correlation coefficients between the number of Lever presses and dLight responses from the same 10 s ITI period analyzed in Fig. 4A. We included trials from the last 2 recorded sessions for each subject (in both sessions all subjects demonstrated higher average press rates during the ITIs relative to the CS interval, Supplementary Fig. 1). A correlation between these measures was only observed for 1 of the 8 subjects suggesting that DA tone is higher during the ITIs independent of the amount of lever press activity.

Given reports that striatal dopamine release may correlate not with general amount of activity, but with the initiation of motor actions, we sought to determine if the dopamine encoding of reward availability was mediated by response initiations. Aligning the dLight signal to lever presses or head entries into the reward port during S^−^ presentations (periods uncontaminated with reward-evoked DA fluctuations), revealed DA transients associated with these actions. On average, the positive DA transients associated with the first lever press in a bout of lever presses (defined as the first lever presses after at least a 2 s pause in pressing) started to increase just prior to the lever press started and peaked shortly after the first press (Supplementary Fig. 10a). These small magnitude transients are concordant with the observation that a minority of VTA DA neurons display phasic excitation just prior to the onset of self-initiated movements^25,11^ which would presumably result in DA release in the NAc, as we observe. In contrast to a positive transient prior to lever presses, a weak negative transient was sometimes observed after head entries into the reward port that occurred during the S^−^ presentations (Supplementary Fig. 10b). Both types of transients were smaller in amplitude than transients observed at S^−^ onset and offset and importantly, smaller than the difference between S^−^ and ITI plateau DA levels. The size and shape of these transients were slightly different for each subject. However, these features remained constant across training for each subject (Supplementary Fig. 10). Thus, dopamine fluctuations related to the initiation and execution of individual actions cannot explain the DA encoding of reward availability states that evolve with training.

Together, these analyses show that while rewards and activity may influence DA level on some scale, DA level (measured by dLight) is lower during the conditioned S^−^ than during the ITI—even when rewards are not presented and is independent of the amount of reward directed activity.

### Dopamine release reflects reward availability before behavioral conditioning

**Fig. 5:**
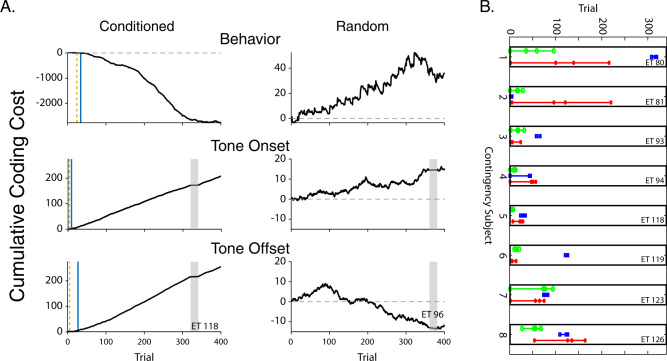
Dopamine reflects reward availability before behavioral conditioning. **A** Cumulative coding costs as a function of trial number are presented for one subject in the conditioned group (Left column) and one subject in the random group (Right column). Cumulative coding costs are depicted for behavior (Tone/ITI lever press ratio) in the top row, DA release (dLight) at Tone Onset in the middle row and DA release at Tone Offset in the bottom row. Note the differences in Y axes scales for the conditioned and random subject. Gray shaded region indicates missing recording session. Vertical dashed line indicates when differences began, vertical solid line indicates significance at *α* = 0.01. **B** The trial number at which 4 events are detected for each variable for each subject in the conditioned group: the beginning of the accumulation of evidence for a difference in the value (in the predicted/ terminal direction) and, 3 levels of significance: odds of 10:1 (*α* = 0.1), 20:1 (*α* = 0.05), and 100:1 (*α* = 0.01). Green = DA at S^−^ onset, Red = DA at S^−^ offset, Blue = behavior.

Long-standing theories about the role of DA in reinforcement learning are founded on the idea that fast transient (phasic) events serve as teaching signals that corrects behavior toward optimal performance on a trial-by-trial basis. Therefore, we sought to determine the temporal relationship between the appearance of the DA transients that occurred at the onset and offset of the conditioning stimulus and the emergence of behavioral conditioning. Because behavioral data and dLight data have different degrees and structures of noise, we sought a method where change is detected in the context of the noise within each specific data stream (behavior or dLight signal). We employed a highly sensitive method for detecting change based on cumulative coding costs (as detailed in the methods section). For the behavioral data, we identified when the behavior during the tone became different from the behavior during the ITI. We did this by comparing the evolving distribution of the intervals between lever presses in the CS (S^−^ or S^0^, for conditioned and control mice, respectively) and ITI periods. On a trial-by-trial basis, these distributions were converted into cumulative parameter estimates of the rates of responding using a Jeffreys prior. We then used the information-theoretic statistic, Kullback–Leibler divergence^26,27^, to determine when (on which trial), the distributions of lever presses in the CS and ITI diverged. Specifically, we identified the first trial on which the cumulative coding cost had a negative sign (i.e., when the estimated rate of responding during the CSs is less than during the ITIs.) and considered this to be the estimate of the trial on which conditioned responding first emerged. We also identified the trial on which the negatively signed cumulative coding cost became permanently less than a criterion of *α* = 0.01. For the dLight photometry data, we first generated CS onset and offset DA trace templates by averaging segments of the DA traces from the last 200 trials for each subject. The onset template was the average for the last 200 trials of the 1.6 s segment immediately following CS onset. The offset template was the average for the last 200 trials of the 1.6 s segment immediately following CS offset. We then correlated the same 1.6 s segments of DA traces for each individual trial to the relevant (CS onset or offset) subject-specific template. We again used Kullback–Leibler divergence to determine the trial on which the correlation between the single trial DA trace and the template diverged from 0. This analysis revealed that DA transients at S^−^ onset and at S^−^ offset appeared before change in behavior in 7/8 conditioned mice (Fig. 5A left column and Supplementary Fig. 11). Typically, the S^−^ onset transients appear before the S^−^ offset transients, reflecting that loss of reward availability is encoded before the return to reward availability is encoded. No changes or even trends for changes in behavior were detected in any of the mice in the random group (Fig. 5A right column and Supplementary Fig. 12). For each subject, DA encoding of the transition in reward availability state preceded behavioral adaptation to this information by a different amount ranging from tens to hundreds of trials (Fig. 5B and Supplementary Fig. 11). These results suggest that the DA transients are not good predictors of the behavioral evidence for learning, providing a further demonstration that DA transients were temporally uncoupled from performance.

### Dopamine dynamics predict the response to unexpected rewards

**Fig. 6:**
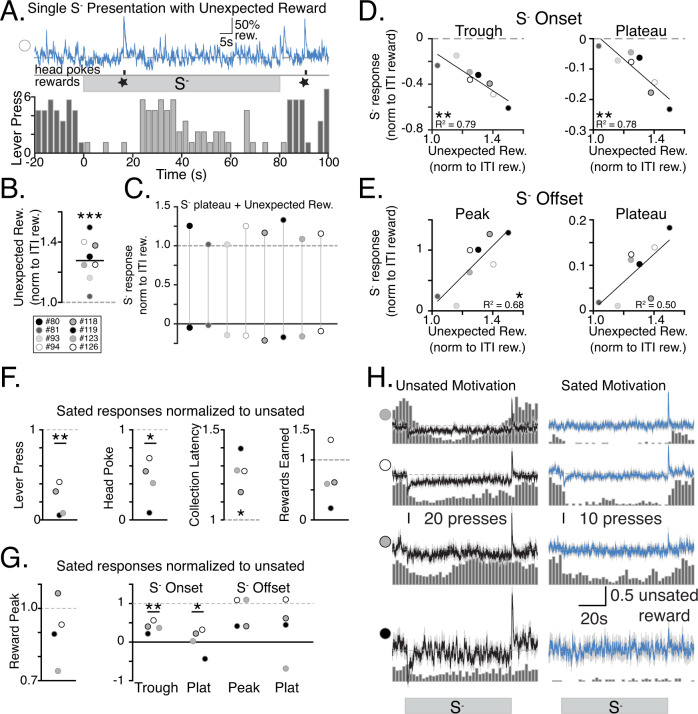
Dopamine dynamics predict response to unexpected reward and are altered by motivational state. **A** Single S^−^ presentation with unexpected reward to lever press during tone. DA response on top and behavior below. The dLight trace is scaled proportional to 50%rew(ard), 50% of the average peak response to reward on that day of recording. **B** Average peaks of unexpected rewards normalized to rewards earned during ITI. One-sample *t* test compared to 1.0: *t* (7) = 5.499, *p* = 0.0009. **C** The DA response to surprising rewards during S^−^ (relative to ITI rewards) is depicted for each subject by a line that extends from the S^−^ plateau (below 0) to its peak height relative to ITI baseline levels. **D**, **E** Strength of encoding of S^−^ onset (**D**) and offset (**E**) compared with amplitude of unexpected rewards. Linear Regression: Extra Sum of Squares F-test for unexpected reward and S^−^ onset trough: *F* (1, 6) = 22.06, *P* = 0.0033, S^−^ onset plateau: *F* (1, 6) = 21.29, *p* = 0.0036, S^−^ offset peak: *f* (1, 6) = 12.58, *p* = 0.0121, S^−^ offset plateau: *f* (1, 6) = 5.922, *p* = 0.0509. **F** Behavior and reward responses following reward satiety normalized to responses with satiety intact, One-sample *t* test compared to 1.0, Lever presses: *t* (3) = 8.659, *p* = 0.0032, Head pokes in reward port: *t* (3) = 4.424, *p* = 0.0214, Collection latency: *t* (3) = 5.559, *p* = 0.0115, Rewards earned: *t* (3) = 1.318, *p* = 0.2792. **G** DA responses to Reward and S^−^ following satiety. One-sample *t* test compared to 1.0, Reward Peak: *t* (3) = 1.39, *P* = 0.2588, S^−^ onset trough: *t* (3) = 8.61, *P* = 0.0033, S^−^ onset plateau: *t* (3) = 5.757, *p* = 0.0104, S^−^ offset peak: *t* (3) = 1.26, *p* = 0.2999, S^−^ offset plateau: *t* (3) 1.657, *p* = 0.1961. **H** Average traces of DA in response to S^−^ presentation with unsated (left) and sated (right) motivation overlaid on histogram of lever presses, dLight traces are scaled proportional to 0.5 rew(ard), where 1 reward is the average peak response to reward for each day of recording. Note difference in scales for lever presses. **B**, **H** gray shading is s.e.m. **B**–**E** *N* = 8 animals. **F**–**H** Sated responses normalized to unsated responses. *N* = 4 animals. **B**, **F**, **G** paired *t* test. **D**, **E** linear regression. *p < 0.05; **p < 0.01. Source data for (**B**, **D**–**G**) are provided as a Source Data file. The raw data used to generate all other figures are deposited in OSF.

The fact that DA levels remained stably depressed during the S^−^ and unaffected by within-trial changes in the level of behavioral action raised two questions: Is DA cell activity and DA release locked in an inhibited state during the S^−^ and unable to change, including in response to reward? And if DA release is not completely locked, is the amplitude of release events affected by the background DA level? To address these questions, we ran a modified version of our task after mice had completed a minimum of 20 regular sessions. We probed the conditional state of DA neurons by rewarding some lever presses during the S^−^. In two sessions separated by a regular training day, selectively during a semi-random subset of S^−^ presentations (6 of the last 15 trials in the session), a single lever press occurring an average of 20 s after S^−^ onset was rewarded. Because the “surprising” rewards were delivered at random, rather than fixed intervals from the S^−^ onset, we cannot display a trace for these events that is averaged across trials. Instead, we provide an example single trial (Fig. 6A) and analyzed DA responses relative to each subjects’ response to the typical rewards earned during the ITI. Transient dLight DA responses to rewards earned during S^−^ occurred and, consistent with RPE signals, were greater in amplitude than transient DA responses to rewards earned during the ITI (Fig. 6A–C and Supplementary Fig. 13; the second session was analyzed as some animals missed collecting many of the unexpected rewards that were delivered in the first test session.

The increase in amplitude to “surprising” rewards (unpredicted rewards delivered during the conditioned state of no reward availability) compared to the ITI rewards that were also unpredicted but presented during reward availability state, correlated with the size of the transients (troughs and peaks) and DA plateau levels associated with S^−^ onset (Fig. 6D) and offset (Fig. 6E). This suggests that the amplitude of phasic events is related to the background plateau level of DA. Indeed, plotting the average change in DA from the S^−^ plateau to the peak response to surprising reward for each subject (Fig. 6C) reveals that approximately half of the difference in DA evoked by reward in the S- vs. the ITI is due to the lower level of DA at the time of reward during the S^−^. Therefore, DA cells are clearly not locked in an inhibited state during the S^−^ and the difference in absolute size of reward-evoked DA transients across reward availability states, indicative of RPE, is at least partially, determined by the state dependent DA tone.

### Motivational state influences DA encoding of changes in reward availability asymmetrically

Lastly, as goal directed behaviors are sensitive to motivational state, we used the same dLight expressing mice to ask whether DA encoding of reward state was influenced by the level of motivation for the reward. We provided unlimited access to reward for 1 h prior to a training session. Sated animals consistently displayed a decreased amount of lever pressing (Fig. 6F) and diminished DA encoding of the S^−^ including a lower amplitude transient dip at onset and a less deep plateau during the S^−^ (Fig. 6G, H). In contrast, changes in DA encoding of S^−^ offset and the DA plateau level during the ITI were inconsistent across subjects (Fig. 6G, H). Therefore, a negative transition to a condition in which rewards are unavailable is sensitive to motivational state, but a positive transition to a condition of reward availability can still be positively encoded even when motivation for the reward is reduced.

## Discussion

Optimal behavior requires adapting to cues that indicate whether it is the right or wrong situation in which to perform a particular action. Many features of reinforcement driven cue-learning are encoded by striatal DA, but whether DA encodes cues that inhibit ongoing effortful reward seeking was previously less explored. Additionally, there are some aspects of reinforcement learning that are not extensively studied. How do conditioned cues of relatively long durations affect DA level? What is the relationship between rapid cue- or reward-evoked DA transients and slower tonic changes in DA that are correlated with motivation?

Here we used an 80 s long negative discriminative stimulus to signal to mice that are working for rewards that rewards were temporarily unavailable. The cue was presented at unexpected times but was fixed in duration so that its termination could be anticipated^28,29^. This allowed us to identify DA signals related to ongoing reward availability states as well as signals related to changes in those states that either were or were not predictable. Using a combination of recordings utilizing FSCV, GCaMP and dLight, we continuously monitored DA release as well as activity in the soma and dendrites of mesolimbic DA neurons.

**Fig. 7:**
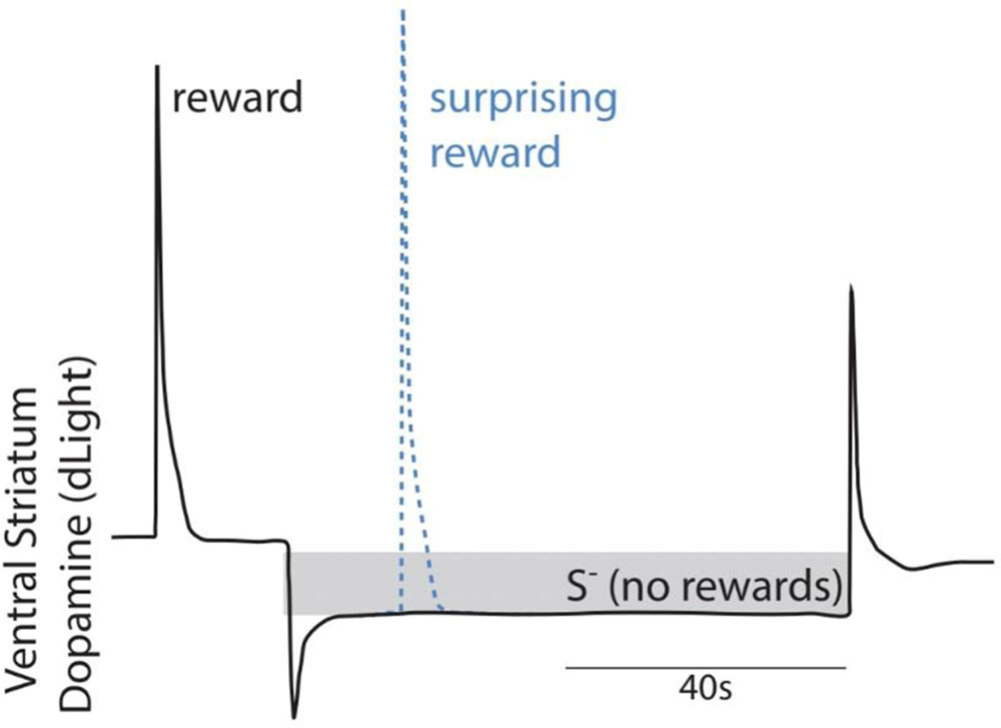
Graphical summary of results. In the ventral striatum abrupt transitions in reward availability states evoke signed dopamine transients. Dopamine tone is persistently lower in periods when rewards are unavailable (S-, no rewards), compared to when they are available. When surprising rewards are delivered in the non-rewarding period, the reward-evoked dopamine transients are larger, even though they arise during a lower background level of dopamine.

Our results are graphically summarized in Fig. 7. We determined that on different timescales, extracellular DA in the ventral striatum encodes reward availability states and the transitions into and out of those states. When rewards are unavailable there is a sustained reduction in DA tone relative to when rewards are available and transitions between states are marked by phasic DA events. During the S^−^, DA remained at a consistently flat low level throughout the fixed-duration S^−^. In other studies, a ramping of DA cell activity over several seconds as reward approaches has been observed (refs. ^30–32^). Such ramps are driven by a progressive increase in the rate of tonic (non-burst) DA cell firing^31^. Reward expectation associated DA ramps are consistent with the temporally discounted value of future rewards though there is ongoing debate if they represent reward state (motivational value)^21,22,33^ or the temporal difference in reward expectation over time^30^. There are at least 3 reasons why we did not observe ramping of mesoaccumbal DA cell activity or dopamine release during the S^−^. First, even though the S^−^ cue is of fixed duration, which makes its termination predictable^34^, lever press rates do not appear to gradually increase within a single S^−^ trial (i.e., a ramping pattern of pressing within a trial is not observed in Fig. 3B). Instead, the time at which a subject resumes lever pressing varies by session, but the probability of resuming lever pressing increases during the S^−^ (Fig. 3C, H). Therefore, while the behavior is sometimes influenced by the anticipation of the return to reward availability, the relationship is not systematic or consistent. Second, in our paradigm, the animal can learn to anticipate when the opportunity to earn a reward will return, rather than when reward will be delivered. Third, our task provides no sensory stimuli or feedback to indicate progress toward reward (e.g., changing cues as the goal is approached). Instead, mice must use an internal representation of the passage of time to predict the S^−^ offset. Previous studies have shown that when rewards are delivered at fixed intervals without dynamic sensory inputs, DA neuron activity does not ramp with expectation (ref. ^30^). Only when movement in space (with or without visual feedback) is required to progress toward reward is DA ramping observed^30,32^.

In a recent study, it was suggested that sustained, motivation-related fluctuations in striatal DA result from locally regulated axonal release^22^. We did not find evidence of such local regulation in our paradigm. In the present study, DA neuron somatic activity in the VTA, DA neuron axonal activity in the ventral striatum were both concordant with each other and with patterns of DA release measured using FSCV and dLight. Because our DA neuron data was obtained using a retrograde GCaMP targeting approach, we are certain that our activity monitoring occurred in cell bodies and terminals within the same mesolimbic projection neurons. Using a similar retrograde labeling approach, Kim et al. also observed the activity of VTA DA neurons was responsible for the changes in calcium signals in DA axons in the ventral striatum^30^.

A surprising finding in the present study is the manifold disconnection between DA and performance. DA encoding of reward availability is not dependent on reward or activity-related fluctuations in DA. DA did not reflect behavioral output at the level of single trials, it remained flat throughout S^−^ irrespective of changes in lever pressing that occurred during the S^−^ demonstrating that action and overall DA tone were not correlated in this paradigm. Furthermore, averaging DA values across trials within a session for all trials in a session DA changes and the pattern of within-trial performance were also not correlated across days. The tonic lower level of DA during the S^−^ is extremely stable across sessions and the size of phasic DA signals at transitions systematically increases across days, yet, behavior continues to show day-by-day variation. The emergence of changes in DA and changes in behavior were also uncoupled. DA transients at S^−^ onset and offset were detected in most subjects before behavioral adaptation and the lag between DA encoding and the reduction in responding during the S^−^ was extremely variable across subjects. Overall, behavior becomes more organized, based on the ratio of lever presses in S^−^/ITI) with training^14^. However, on some days subjects make many anticipatory responses during the S^−^. For example, while the subject depicted in Fig. 3C exhibits excellent efficiency of behavior on some days (6–9, 18–20), there is distinctly less inhibition of behavior during the S^−^ on other days. These session-based differences were unrelated to the DA encoding of cue, cue transitions, or the rewards earned during the ITI and therefore represents an aspect of behavioral control that does not engage mesolimbic dopamine. In our paradigm, long duration cues result in widely spaced periods of reward availability. When rewards are presented periodically sequences of natural behavior consistently emerge^35–37^. These natural behaviors can compete with the goal directed behavior that earns a reward. These natural behaviors are sometimes referred to as interim behavior^36^ or instinctive drift^38^. Speculatively, trial-to-trial variation in these evoked sequences may give rise to the variation in bar pressing. In the conditioning chamber, natural behaviors such as exploration, grooming or unconditioned interaction with response operandi^37,39^ might be evoked during the S^−^ periods. Such behaviors may reflect factors that vary from day to day, not be regulated by DA and have a large impact on the pattern of responding during the S^−^.

If the DA encoding does not reflect movement or support performance, what is its purpose? One function of the observed shifts in DA tone may be to serve as an internal representation of reward availability, which, along with other factors such as motivation for the specific reward type and effort requirement sets the relative background upon which phasic DA events are superimposed. Indeed, when we provided surprise rewards for lever pressing during the S^−^, reward-evoked DA was greater than during the ITI. This was the case even when the relatively lower DA background was taken into account. Therefore, the increased size of phasic DA responses evoked when rewards are unexpected arises from two aspects of DA dynamics. The phasic response emerges from a lower tonic level of DA when reward is not expected and the peak phasic response is of greater magnitude, compared to when reward is expected. Both factors contribute to the magnitude of the DA RPE.

Our results are also consistent with the concept that internal state can rescale DA signals. When mice were sated prior to testing, the difference in steady-state levels of DA between reward availability states was diminished. Cue and reward-evoked DA transients were also reduced by pre-session sating. Therefore, we determined that, as for excitatory food predictive cues^40,41^, satiety attenuates the DA response to negatively predictive food cues.

More generally, our results highlight the idea that learning about positive or negative conditioned stimuli always involve learning about both. The positive predictive value of any cue depends on there being predictive periods of time during which no outcome is presented^13,14^. The excitatory value of a cue depends on the animal learning when outcomes do not occur as well as when they will be presented. Similarly, the value of an inhibitory cue depends on the animal learning when outcomes will and will not occur. They are two sides of the same coin. Here we show that DA encodes both these aspects of contingencies in a symmetric fashion. Both the transient and steady-state DA response reflect the information that cue onset and offset provide about the availability and non-availability of reward^14^. It will be interesting to learn how each of the components of the DA response contributes to learning.

With respect to DA, our results show that cues which inhibit responding are encoded by DA tone, and this tone serves to provide the relevant contrast for phasic events. As previously suggested by others^21,30^ our study shows that consideration of the DA context, often referred to as “baseline”, is important for understanding the relevance of fast dynamic DA signals.

## Methods

### Animals

All animal studies were approved by the New York State Psychiatric Institute Animal Care and Use Committee and were performed in accordance with PHS Policy on Humane Care and Use of Laboratory Animals.

Male C57BL/6J wild-type mice were purchased from Jackson Laboratories. DAT-ires-cre mice (B6.SJL-Slc6a3tm1.1(cre)Bkmn/J) were purchased from Jackson Laboratories and bred in our animal facility to produce Male and Female heterozygous mice for experiments. Mice were housed singly or in groups of up to 4, maintained on a 12:12 light:dark cycle and tested during the light phase. Temperatures are maintained in a range of 22 °C ± 2 ^°^C. Relative humidity is maintained between 30 and 70%. Mice were a minimum of 10 weeks of age at the start of experiments. Mice were food restricted to maintain their weight at 85% ad lib weights. No sample size precalculation was performed.

### Behavior

#### Apparatus

Behavioral pretraining and training were performed in experimental chambers with a single lever to the left of a feeder trough centered on one side of the chamber (Med-Associates). A house light was placed to the side of the chamber within the enclosure to allow for removal or adaption of chamber ceiling to accommodate a tether for recording. Each enclosure had an exhaust fan which provided background white noise of ~72 dB. Speakers delivered the S^−^ at 3.5 kHz and 80 dB and were positioned on the wall opposite the lever and the food port.

#### Behavior paradigm

Animals were pre-trained to press a lever initially on a continuous reward schedule and then on a random interval schedule of an average of 5 s, 10 s, and finally 20 s between each reward (as described previously,^14^). Animals were rewarded with 20 µL of evaporated milk. Training commenced after animals consistently earned 40 rewards on a random interval (RI) 20 schedule in less than 30 min. For the conditioned group, a training session included 20× 80-s-long presentations of the tone with an average intertrial interval (ITI) of 40 s (range 1.5–90 s, drawn from an exponential-like distribution). In between tone presentations (during the ITI), rewards could be earned on an RI 20 s schedule. For the random group, training sessions similarly included 20× 80-s-long presentations of a tone. Rewards could be earned at any time during the session, including during the tone, on an RI60 schedule, which was chosen so that both groups of animals could earn similar numbers of rewards across each session. In a subset of animals, after at least 20 training sessions, DA responses to unexpected rewards were probed by rewarding a lever press during a conditioned stimulus in 6 of the final 15 tone presentations after an average of 20 s from tone onset. After these probe sessions, some animals were trained on the original paradigm for 5 more sessions and then sated on reward (evaporated milk) for 1 h prior to the training session.

### Voltammetry

Adult male C57BL/6J mice (8 weeks or older) were bi-laterally implanted with carbon-fiber microelectrodes constructed as described^42^. Briefly, a carbon-fiber encased in a polyimide fused silica is electrically insulated by applying a two-component epoxy to the fused silica carbon-fiber interface. At the opposite end, a female pin connector is electrically connected to the carbon fiber with silver epoxy. Finally, two-component epoxy is used to coat the connector for electrical insulation and structural integrity. The response of microsensors constructed this way is linear to physiological concentrations of dopamine^42^. Electrodes were positioned in lateral nucleus accumbens (NAc; AP + 1.2 mm; ML 1.5 mm; DV −4.0 mm). Animals recovered for 2–3 weeks before starting behavior and recordings began at least 4 weeks post-surgery. For recordings, microelectrodes were connected to a head-mounted voltammetric amplifier (Scott Ng-Evans, University of Washington) for dopamine detection using fast scan cyclic voltammetry. Triangular waveforms sweeping from −0.4 to 1.3 V were applied at 10 Hz and controlled by custom software (TarHeel CV), written in LabView (National Instruments, Austin, TX).

#### Analysis

To extract the DA component from the voltammetric recordings we obtained the DA oxidation current using background subtraction and chemometric analysis. For DA traces aligned to specified events (Rewards, cue onset and offset, Fig. 1C, D, F) background subtraction was set 0.5 s before the event. To visualize DA traces across an entire trial (10 s before and 10 s after the 80 s cue, Fig. 1E) background subtraction was set at the midpoint of the S^−^). We used principal component regression against a training set of electrically evoked DA and pH cyclic voltammograms with two principal components^43,44^. DA concentration was obtained from the calculated DA currents using a calibration factor of ~80 nA/mm. This factor was based on a dataset developed in vitro to quantify DA oxidation current versus nonfaradaic background current using the method of^45^. To allow us to combine voltammetric data from different subjects and/or across different sessions (which may be subject to day-to-day variation in signal recovery), DA responses to the S^−^ were normalized to the average peak response to reward for each day for each recording.

#### Histology

Probe placement in NAc was confirmed by making a small electrolytic lesion at the tip of the microelectrode under anesthesia. Brains were quickly removed and flash-frozen in isopentane (Sigma aldrich) cooled to −20 °C. Tissue was cryosectioned at 50 µm and sections were inspected under a light microscope.

### Photometry

Photometry experiments were performed in adult male and female DAT-ires-cre heterozygous mice on a C57BL/6J genetic background. Mice were at least 8wks old at the time of surgery. Viruses encoding either GCaMP6f or dLight were injected into left NAc (AP + 1.2 mm; ML −1.5 mm; DV −4.0 mm). GCaMP6f was retrogradely transported using hEF1ɑ-LS1L-GCaMP6f (150nL; MGH Virus Vector Core). dLight was expressed locally using AAV5-CAG-dLight1.1 (200 nL; Addgene). For GCaMP6f recordings, mice were implanted with 400 µm optical fiber (Doric) in the left hemisphere above either NAc (AP + 1.2 mm; ML −1.5 mm; DV −4.0 mm) or VTA (AP −3.0 mm; ML 0.85 mm; DV −4.2 mm). For dLight recordings, mice were implanted with a 400 µm optical fiber in the left hemisphere above NAc. Animals recovered for 2–3 weeks before behavioral testing and recordings began at least 4 weeks post-surgery. Imaging was performed using optical components (Doric Lenses) controlled by RZ5P acquisition processor (TDT). Two photodiodes (405 and 465 nm) were sinusoidally pulsed (at 210 and 330 Hz, respectively; if two animals were recorded from simultaneously, photodiodes for animal 2 were pulsed at 270 and 450 Hz, respectively). Traces were demodulated online. The times of behavioral variables were recorded as TTL inputs to the acquisition system and through the MED-PC system.

#### Analysis

Data were analyzed using custom Matlab scripts. To correct for potential motion artifact or within session photobleaching, we normalized the GCaMP6f-Ca++ and dLight specific signal generated by 465 nm excitation to the isosbestic signal generated by 405 nm excitation^23^. Specifically, we calculated ΔF1/F2 by taking a least squares linear fit of the 405 nm channel value aligned to the 465 nm channel. Consistent with the FSCV experiments, all traces were background subtracted using a 0.5 s window immediately prior to each event. GCaMP6f and dLight recordings were low pass filtered at 2 and 5 Hz, respectively, based on the kinetics of each sensor. To normalize for signal recovery for each mouse on each experimental day, DA responses to the S^−^ were normalized to the average peak response to reward for each day of recording.

#### Histology

Animals were anesthetized and transcardialy perfused with 4% PFA. Brains were sectioned on a vibratome (50 µm) and underwent immunohistochemistry. Transgenic protein expression was amplified using antibody to GFP (ab13970, Abcam), 1:1000). DA neurons were identified using anti-tyrosine hydroxylase antibodies (AB152 Millipore Sigma), 1:5000).

##### Data analysis

Statistical tests, described in the figure legends, included paired *t* tests, one-way and two-way ANOVA with appropriate treatment of within and between factor variables. Analysis was performed using custom-written Matlab or python scripts.

##### Method for the cumulative coding cost based analysis

To determine the temporal relationship between the appearance of the DA transients that occurred at the onset and offset of the conditioning stimulus and the emergence of behavioral conditioning, we applied a highly sensitive method for detecting change within a data stream based on cumulative coding costs. The cost in bits/datum of encoding a data stream is minimized only when the encoding process uses the most accurate stochastic model of the source statistics (Shannon’s source coding theorem^46^). The Kullback–Leibler divergence, D_KL_ (P||Q),of an inaccurate encoding model, P, from the most accurate model, Q, gives the cost in bits/datum of encoding data coming from Q on the erroneous assumption they come from P^47^ Thus, the cumulative cost of coding *n* data drawn from Q using an inaccurate model P is *n*D_KL_(P||Q).

Given an assumed form for the source distribution (normal, exponential, etc), *n*D_KL_(P||Q) is a function only of the difference between the parameter vectors, **θ**_P_ and **θ**_Q_. Thus, when the data come from an exponential distribution, the divergence depends only on the rate parameters (*λ*_P_ and *λ*_Q_); when they come from a normal distribution, it depends only on the means and standard deviations **θ**_P_ = 〈μ_P_ *σ*_P_〉 and **θ**_Q_ = 〈μ_Q_ *σ*_Q_〉.

For distributions in the exponential family, it can be shown that, on the null hypothesis P ≡ Q, *n*D_KL_(P||Q) is distributed according to the gamma distribution, with shape parameter equal to half the number of elements in the parameter vector of the source distribution, and scale parameter 1. Thus, when the data come from an exponential distribution ${n{{{{{\rm{D}}}}}}}_{{{{{{\rm{KL}}}}}}}\left({\lambda }_{{{{{{\rm{P}}}}}}}{{{{{\rm{||}}}}}}{\lambda }_{{{{{{\rm{Q}}}}}}}\right) \,\sim\, \Gamma \left(0.{{{{\mathrm{5,1}}}}}\right)$ on the null hypothesis; when they come from a normal distribution, ${n{{{{{\rm{D}}}}}}}_{{{{{{\rm{KL}}}}}}}\left({\theta }_{{{{{{\rm{P}}}}}}}{{{{{\rm{||}}}}}}{\theta }_{{{{{{\rm{Q}}}}}}}\right) \,\sim\, \Gamma \left({{{{\mathrm{1,1}}}}}\right)$. For details see Appendix B in ref. ^48^.

Here we used the *n*D_KL_ statistic (cumulative coding cost) to measure trial by trial the strength of the evidence that the behavioral data from periods when the CS was present came from a different distribution than the data from the intertrial intervals. We also used the *n*D_KL_ statistic to measure trial by trial the strength of the evidence of the appearance of a dopamine transient (recorded using dLight and fiber photometry) at the onset and offset of the CS.

##### For the behavioral data

Bar press rates were computed separately for the CS (S^−^ or S0) period (λ_CS_) and the ITI period (λ_ITI_) and any time spent in the reward receptacle was excluded. The distribution of intervals between lever presses were approximately exponential. Our Computed Bayesian estimates of the response-rate parameters during CSs and ITIs as of the *r*^th^ response used the Jeffreys prior, which is the gamma probability density distribution with shape = 0.5 and scale = 0, denoted *γ* (0.5, 0). To obtain the estimate of the cumulative coding cost of assuming that the press rate during the CSs was the same as during the ITIs (the null hypothesis), we integrated out the uncertainties about the values of the rate parameters, *λ*_r|CS_ and *λ*_r|ITI_:

1 $$n{{{{{{\rm{D}}}}}}}_{{{{{{\rm{KL}}}}}}}\left(r\right)\,=\,n\iint {{{{{\rm{\gamma }}}}}}\left({{{{{\rm{p}}}}}}\left({\lambda }_{{{{{{\rm{r|CS}}}}}}}\right)|{{{{{{\boldsymbol{\theta }}}}}}}_{{{{{{\rm{CS}}}}}}}\left(r\right)\right){{{{{{\rm{\gamma }}}}}}\left({{{{{\rm{p}}}}}}\left({\lambda }_{{{{{{\rm{r|ITI}}}}}}}\right)|{{{{{{\boldsymbol{\theta }}}}}}}_{{{{{{\rm{ITI}}}}}}}\left(r\right)\right){{{{{\rm{D}}}}}}}_{{{{{{\rm{KL}}}}}}}\left({\lambda }_{{{{{{\rm{r|CS}}}}}}}{||}{\lambda }_{{{{{{\rm{r|ITI}}}}}}}\right)d{\lambda }_{{{{{{\rm{r|CS}}}}}}}d{{\lambda }_{{{{{{\rm{r|ITI}}}}}}}}_{{{{{{\rm{t}}}}}}},$$

where the integrals run from the first response to the *r*^th^ response, and the **θ**(*r*)’s are the parameter vectors of the gamma posterior distributions on *λ*_r|CS_ and *λ*_r|ITI_, as of the *r*^th^ response. Given these response-by-response estimates of the response rates, our measure of the weight, *W*, of the evidence for a difference in response rates was the common log of the odds against the null hypothesis:

2 $${W}_{{\hat{\lambda }}_{{{{{{\rm{r|CS}}}}}}} \, < \, {\hat{\lambda }}_{{{{{{\rm{r|ITI}}}}}}}}\left(r\right)\,=\,{{{{{\rm{log }}}}}}\left(\frac{p\left(r\right)}{1\,-\,p\left(r\right)}\right),$$

where $p\left(r\right)\,=\,\Gamma \left(n{{{{{{\rm{D}}}}}}}_{{{{{{\rm{KL}}}}}}},0.{{{{\mathrm{5,1}}}}}\right)$ and Γ denotes the cumulative gamma distribution.

Supplementary Fig. 14 depicts the cumulative coding costs for behavior from 2 subjects in the conditioned group that represent extreme performances in terms of speed of learning. Estimates of the rate parameters during S^−^ presentations (*λ*_r|CS_) and ITIs (*λ*_r|ITI_) and the *signed* cumulative coding cost are presented as a function of trial number. It is critical that Cumulative coding costs are signed because D_KL_ ≥ 0, regardless of the direction of the divergence, *n*D_KL_ is always positive. However, the conditioned response in an inhibitory protocol is considered to have appeared only when the estimated rate of responding during the CSs is less than during the ITIs. (Early in training, it is often greater than.) Therefore, we assigned to *n*D_KL_ the sign of the difference between the rate estimates (${\hat{\lambda }}_{{{{{{\rm{r|CS}}}}}}}\,-\,{\hat{\lambda }}_{{{{{{\rm{r|ITI}}}}}}}$). The evidence that the conditioned response had appeared was considered strong only when *n*D_KL_ had negative sign and ${W}_{{\hat{\lambda }}_{{{{{{\rm{r|CS}}}}}}} \, < \, {\hat{\lambda }}_{{{{{{\rm{r|ITI}}}}}}}}\left(r\right) \, > \, 0.52$ (a criterion equivalent to *α* = 0.01). The signed cumulative coding costs for all 8 subjects in the negative contingency condition and all 4 subjected in the zero (random) contingency condition are plotted against trials in the first column on Supplementary Figs. 11, 12 respectively.

##### For the photometric data

The dLight photometric data were approximately normally distributed. We used the Jeffreys prior on the mean and precision of a normal distribution, ${{{{{{\boldsymbol{\theta }}}}}}}_{{{{{{\rm{dL}}}}}}}\,=\,\left\langle {\mu }_{{{{{{\rm{dL}}}}}}}{\tau }_{{{{{{\rm{dL}}}}}}}\right\rangle$, where *τ* = 1⁄*σ*^2^, to obtain Bayesian estimates of the mean and precision of the photometric signal as of trial (*t*). The Jeffreys prior for the normal when it is parameterized by its mean and precision is the normal-gamma distribution with hyperparameters **θ**_ng_ = 〈0 0–0.5 0〉. This prior has the unique property that the estimates of the normal distribution’s parameters are invariant under change of parameter. This property licenses the use of the change of variable formula: ${\hat{\sigma }}_{{{{{{\rm{dL}}}}}}}\,=\,{{{{{\rm{sqrt}}}}}}\left(1/{\hat{\tau }}_{{{{{{\rm{dL}}}}}}}\right)$ to obtain our estimate of the standard deviation.

To measure the extent to which the negative spike in the photometric signal was present at CS onset and the extent to which the positive spike in the signal was present at CS offset, we computed templates for these spikes by averaging over the last 200 trials (Supplementary Fig. 15). The onset template was the average over the 200 1.6 s segments immediately following CS onset. The offset template was the average over the 200 1.6 s-long segments immediately following CS offset. The measures of the extents to which these spikes were present on a given trial were the correlations between the appropriate template (onset or offset) and the corresponding segment of the photometric signal from each trial. These correlations were approximately normally distributed. We estimated the means and standard deviations of these normal distributions trial by trial using the Bayesian procedure already described for estimating the mean and standard deviation of the signal. We computed the strength of the evidence that the mean template correlations were > 0 using:

3 $${W}_{{\hat{\mu }}_{{r}_{{{{{{\rm{CS}}}}}}} \, > \, 0}}\left(t\right)\,=\,{{{{{\rm{log }}}}}}\left(\frac{p\left(t\right)}{1\,-\,p\left(t\right)}\right),$$

where $p\left(t\right)\,=\,\Gamma (n{{{{{{\rm{D}}}}}}}_{{{{{{\rm{KL}}}}}}}({\hat{\mu }}_{{r}_{{{{{{\rm{CS}}}}}}}}(t){{{{{\rm{||}}}}}}{\mu }_{{r}_{{{{{{\rm{CS}}}}}}}}\,=\,0),1,1)$ with the standard deviations for the null distributions equated to the estimate for the CS distribution.

### Reporting summary

Further information on research design is available in the Nature Research Reporting Summary linked to this article.

## Supplementary information

Supplementary Figures

Reporting Summary

## Data Availability

All data generated in this study (Complete Behavior, FSCV, and Photommetry datasets) used to generate the source data and the traces presented in this paper (but not statistically tested) are available on OSF: https://osf.io/d4fuj. Source data are provided with this paper.

## Source data

Source Data File

## Code availability

The code used to run and analyze the behavior, and analyze the voltammetry and Photommetry data is available on OSF: https://osf.io/d4fuj.
