# Supplementary material for: Dopamine encodes real-time reward availability and transitions between reward availability states on different timescales
Source: Nat Commun. 2022 Jul 1;13:3805. doi: 10.1038/s41467-022-31377-2 (PMC9249893; doi:10.1038/s41467-022-31377-2)
Supplement: Supplementary file 1 — Supplementary Figures [file 41467_2022_31377_MOESM1_ESM.pdf]

Supplementary Figure 1

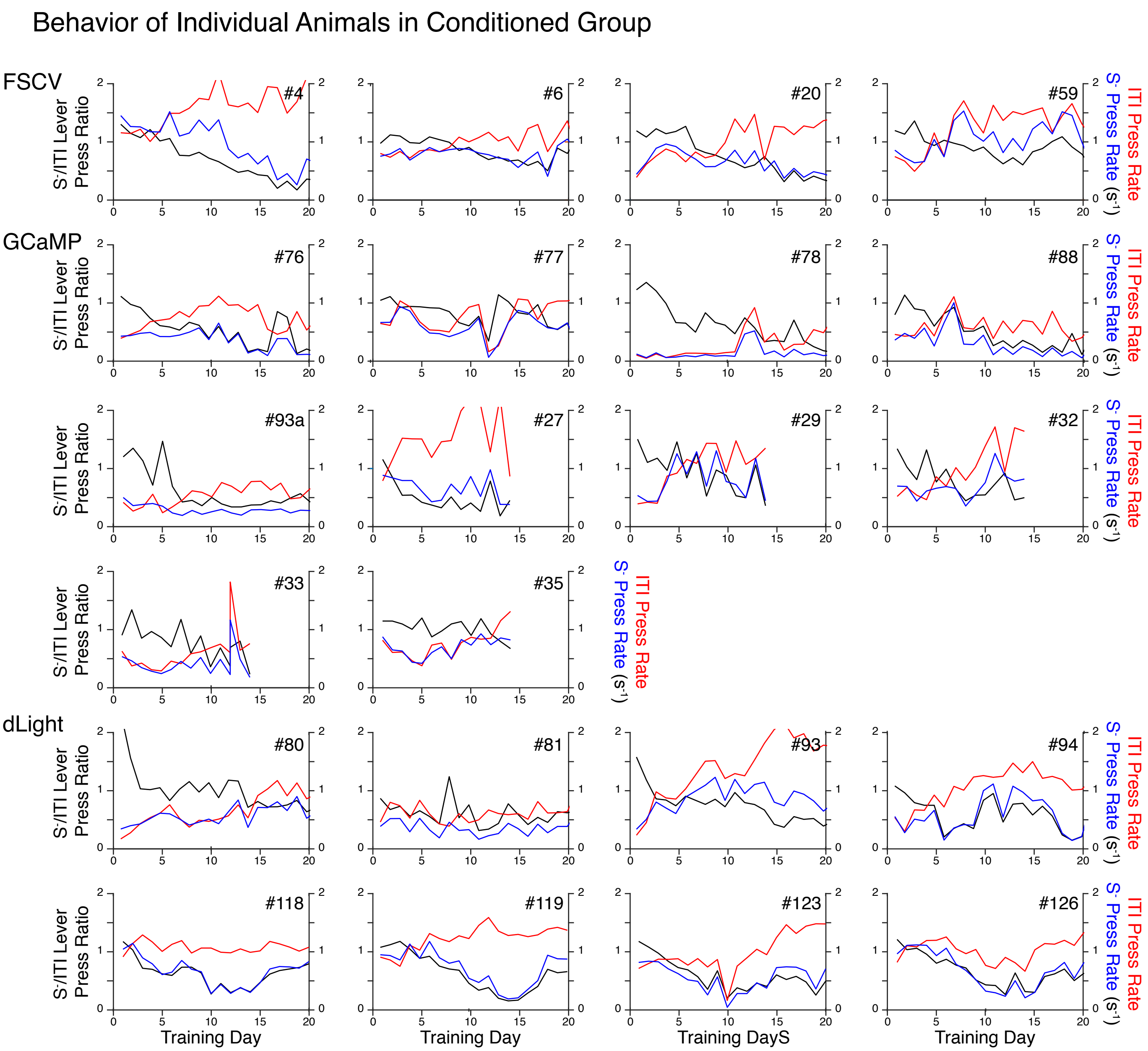

Supplementary Figure 1: Plots of lever pressing over training from animals in Conditioned Group.
Left axis (black traces): S^-/ITI lever press ratio
Right axis: Red traces: ITI press rate; Blue traces: S^- press rate (s^{-1})

Supplementary Figure 2

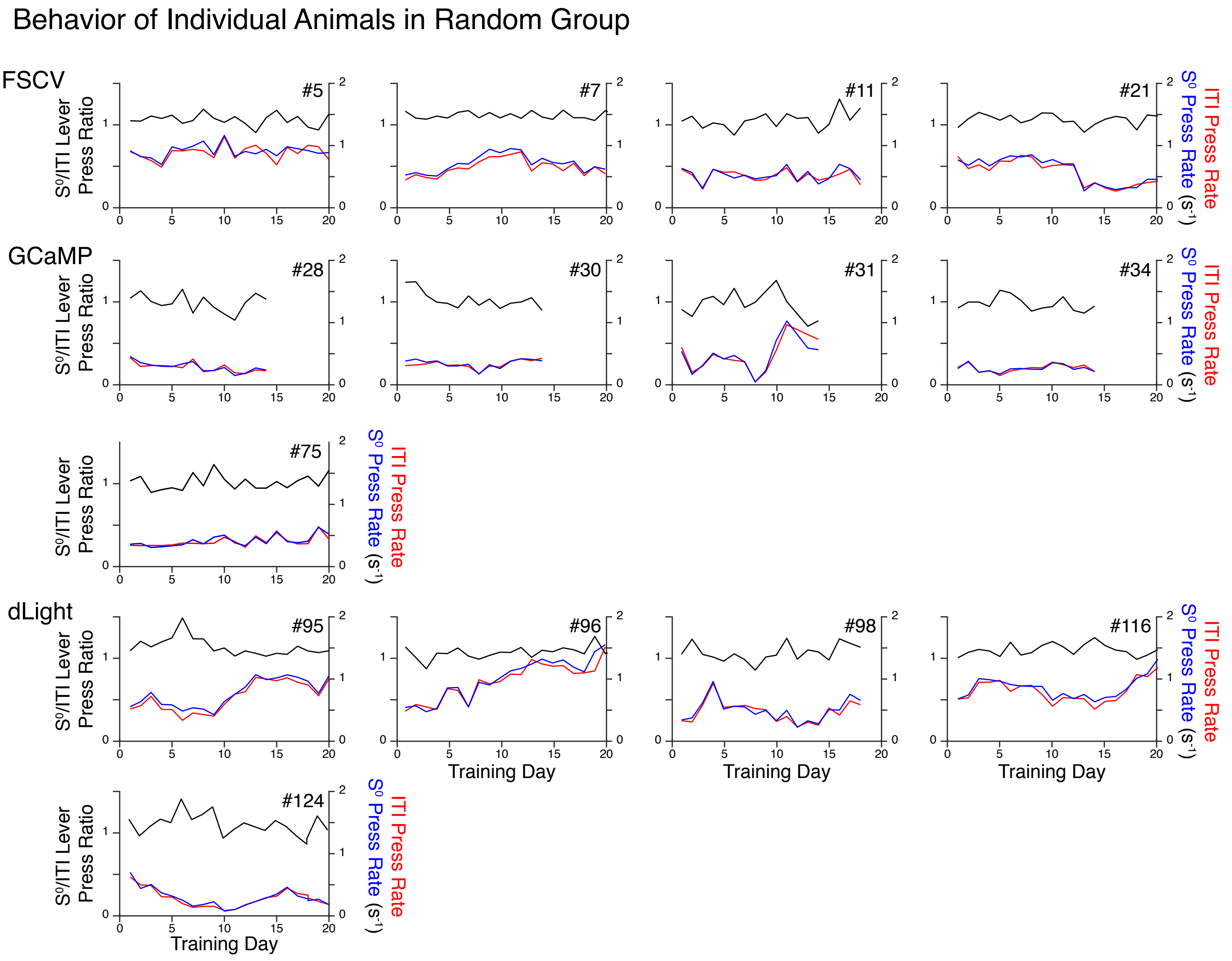

Supplementary Figure 2: Plots of lever pressing over training from animals in Random Group.
Left axis (black traces): S^0/ITI lever press ratio
Right axis: Red traces: ITI press rate; Blue traces: S^0 press rate (s^{-1})

Supplementary Figure 3

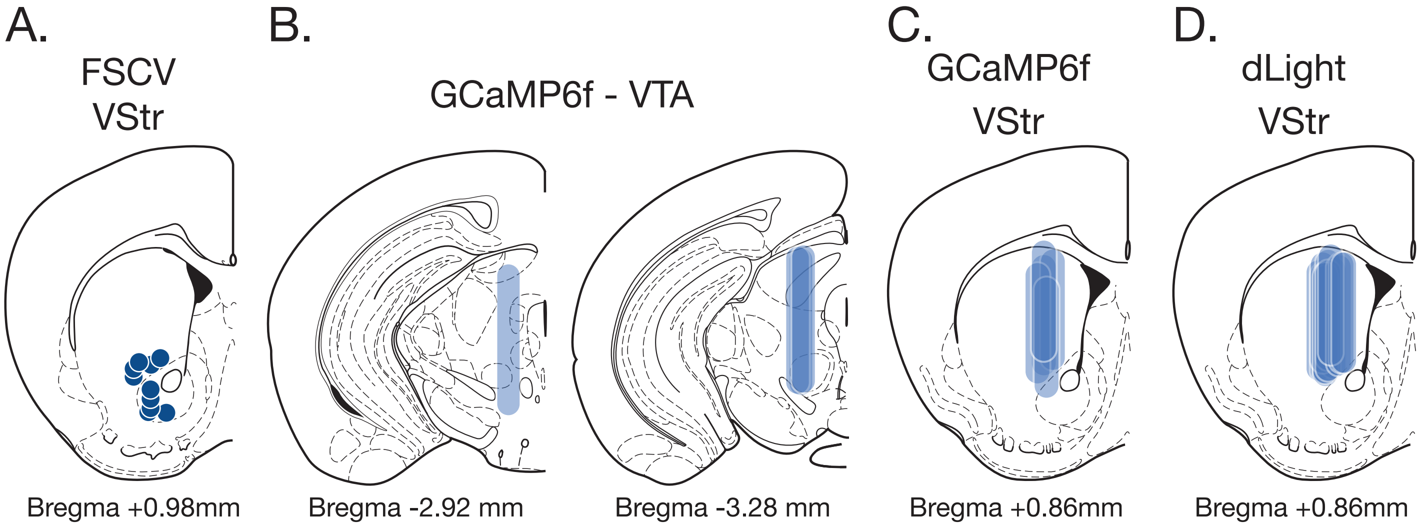

Supplementary Figure 3: Probe locations
A. FSCV probel locations in ventral striatum (VStr), n=11 fibers, N=8 mice
B. Probe locations placed in VTA for GCaMP6f retrograde experiments, n=5 probes, N=5 mice
C. Probe locations placed in VStr for GCaMP6f experiments, n=9 probes, N=9 mice
D. Probe locations placed in VStr for dLight experiments, n=12 probes, N=12 mice

Supplementary Figure 4

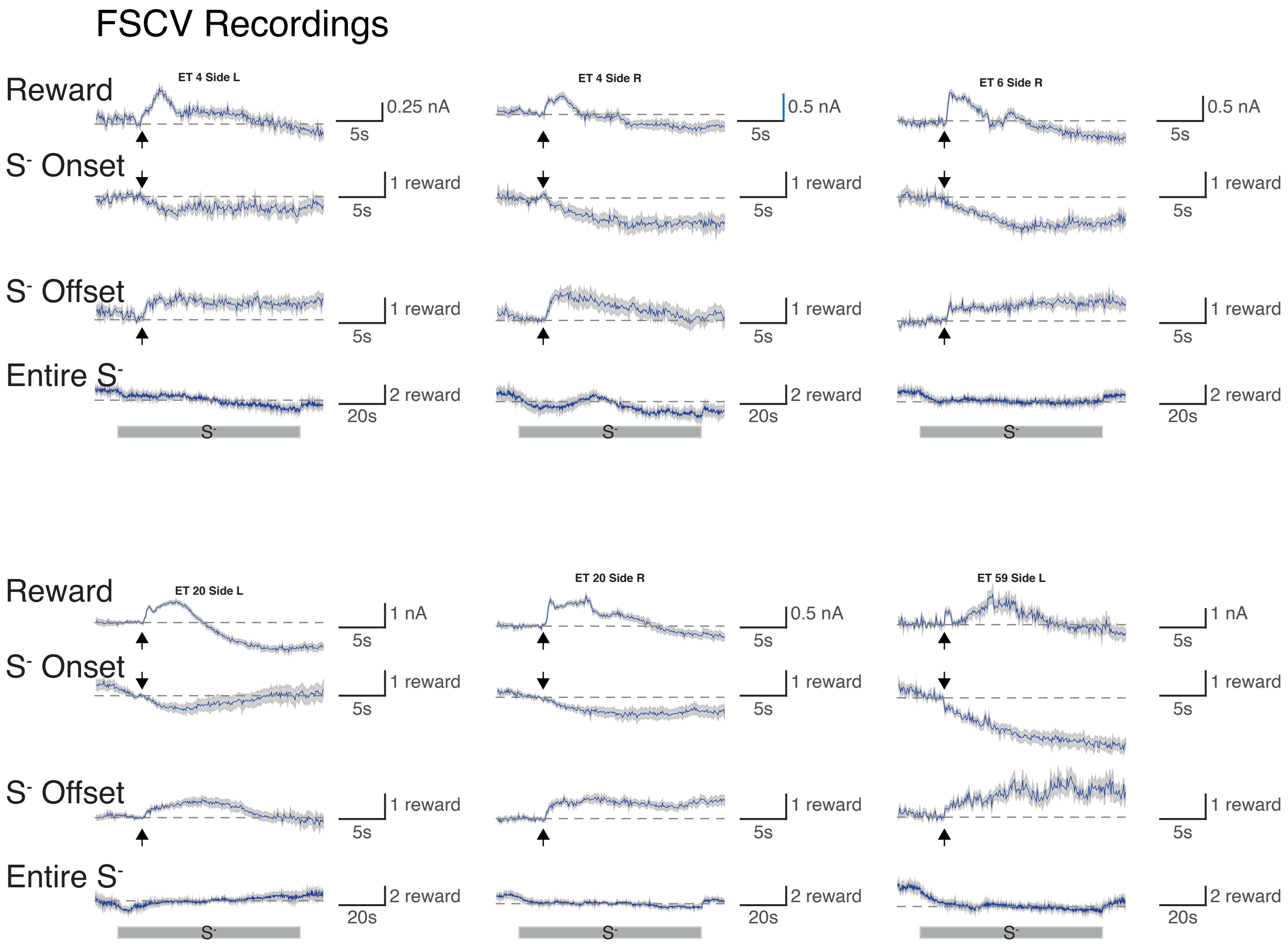

Supplementary Figure 4: DA responses for each FSCV fiber in Conditioned animals.
6 fibers from 4 different animals were included in final data set for FSCV analysis of Conditioned responses plotted in Figure 1. Reward, S- onset, and S- offset indicated with arrows. Entire S- period indicated with grey bar. Responses were background subtracted at 0.5s before arrows (top 3 traces for each animal) or in middle of the S- (bottom trace for each animal). Blue line are averages of all 20 trials in last training session. Grey shading is s.e.m.

Supplementary Figure 5

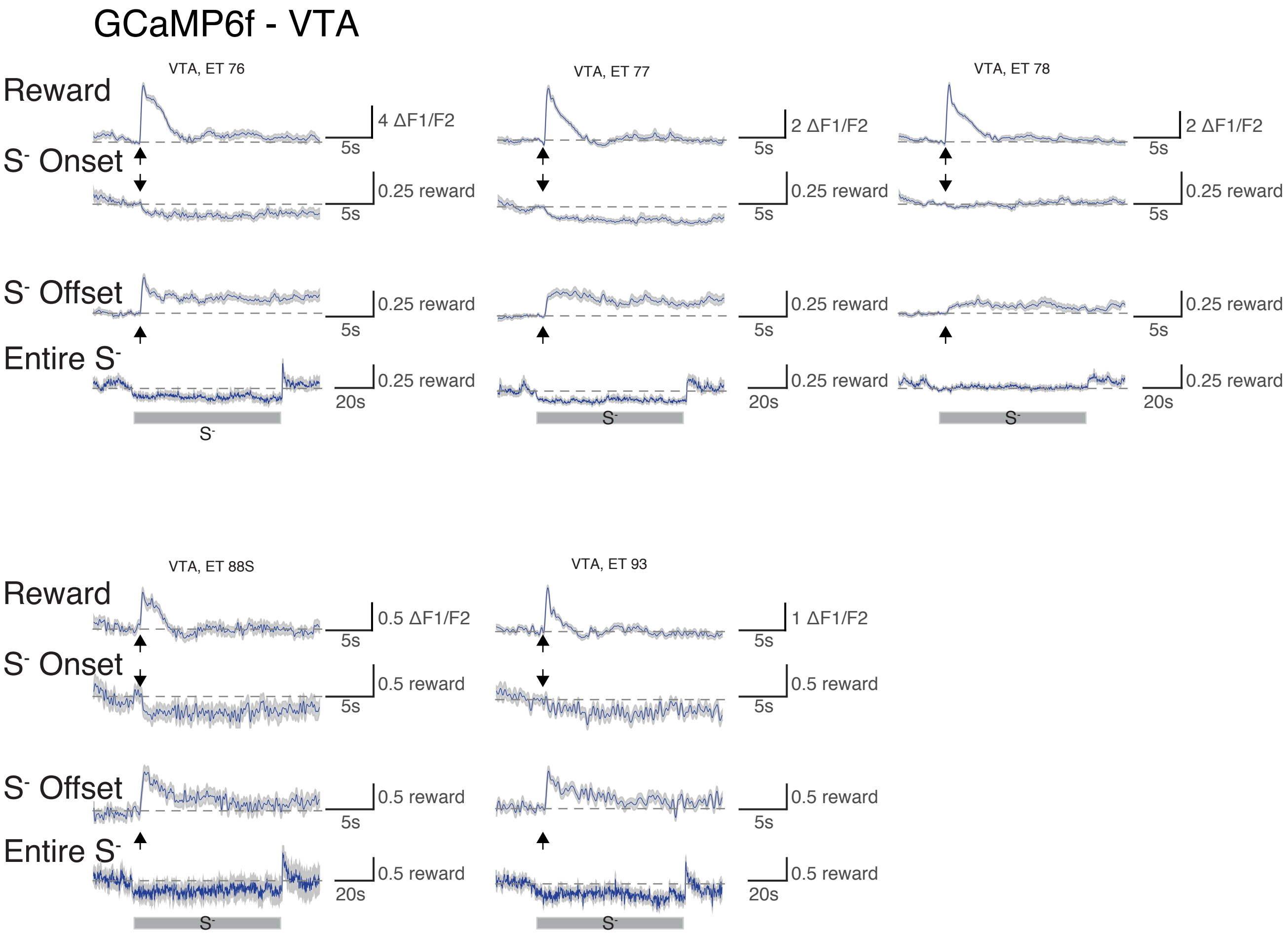

Supplementary Figure 5: DA responses for each optical fiber implanted in VTA of Conditioned animals expressing GCaMP6f in DA neurons

5 fibers each from a single animal were included in final data set for analysis of Conditioned responses measured with GCaMP6f in DA cell bodies plotted in Figure 2. Reward, S- onset, and S- offset indicated with arrows. Entire S- period indicated with grey bar. Responses were background subtracted at 0.5s before arrows or beginning of S-. Responses are average of 20 trials from final training day. Grey shading is s.e.m.

Supplementary Figure 6

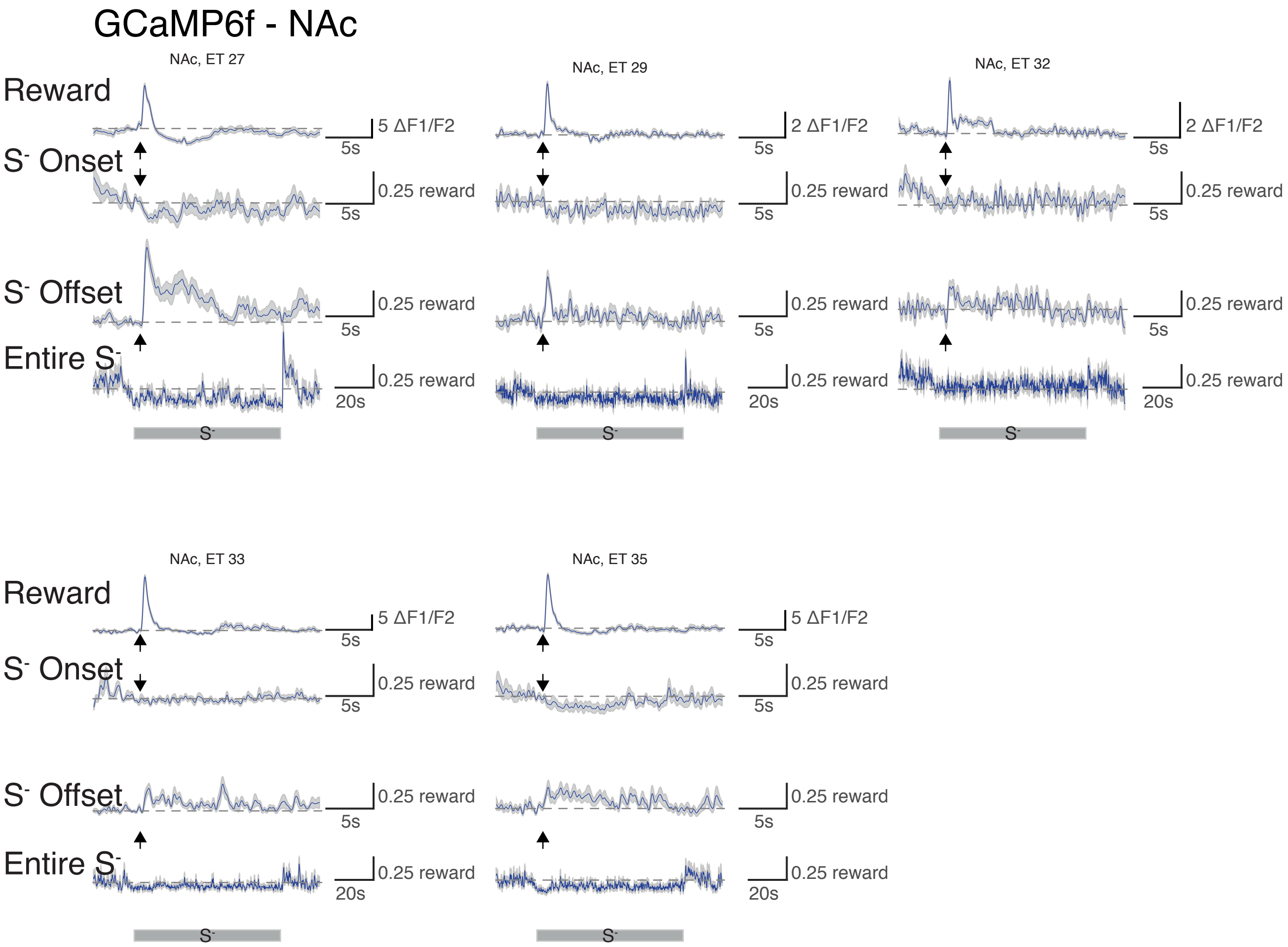

Supplementary Figure 6: DA responses for each optical fiber implanted in NAc of Conditioned animals expressing GCaMP6f in DA neurons
5 fibers each from a single animal were included in final data set for analysis of Conditioned responses measured with GCaMP6f in DA axons plotted in Figure 2. Reward, S- onset, and S- offset indicated with arrows. Entire S- period indicated with grey bar. Responses were background subtracted at 0.5s before arrows or beginning of S-. Responses are average of 20 trials from final training day. Grey shading is s.e.m.

Supplementary Figure 7

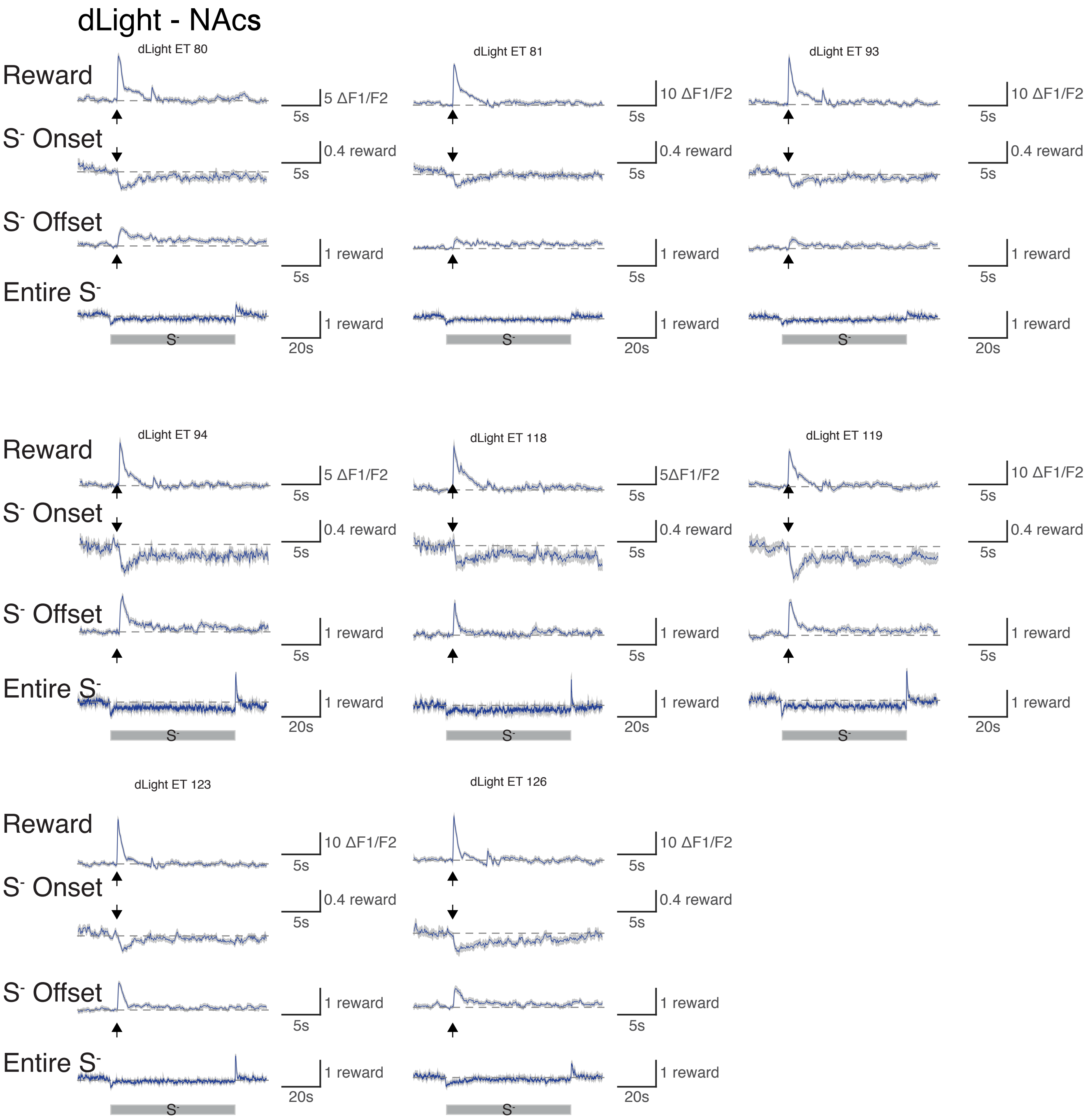

Supplementary Figure 7: DA responses for each optical fiber implanted in NAc of Conditioned animals expressing dLight.
8 fibers each from a single animal were included in final data set for analysis of Conditioned responses measured with dLight plotted in Figure 2. Reward, S- onset, and S- offset indicated with arrows. Entire S- period indicated with grey bar. Responses were background subtracted at 0.5s before arrows or beginning of S-. Responses are average of 20 trials from final training day. Grey shading is s.e.m.

Supplementary Figure 8

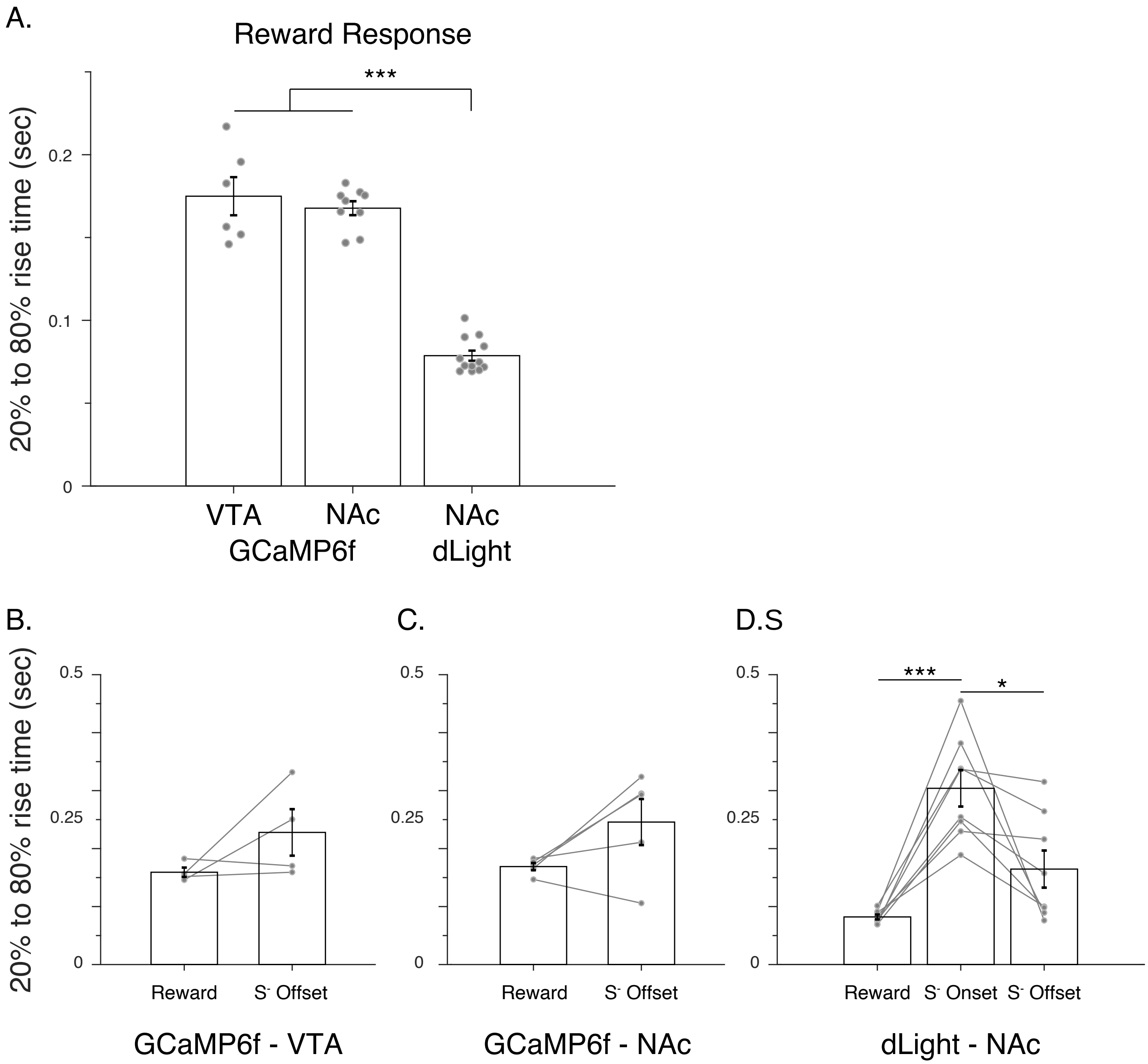

Supplementary Figure 8: Rise times depend on indicator but not recording location.
A. Rise time of response to reward (dipper presentation) was defined at the time elapsed between 20% and 80% of maximum amplitude. Rise time was not different for GCaMP6f recordings of DA cell bodies in the VTA versus their axons in NAc. However rise times are much faster with dLight indicator. One-Way ANOVA: $F(2, 24) = 104.8$, $P<0.0001$. Tukey's multiple comparisons test: GCaMP VTA vs. NAc: Mean Diff 0.0072, 95% CI of diff. -0.01439 to 0.02883, P = 0.6856. GCaMP VTA vs. dLight NAc: Mean Diff 0.0963, 95% CI of diff. 0.07576 to 0.1168, P = <0.0001. GCaMP NAc vs. dLight NAc: Mean Diff 0.0890, 95% CI of diff. 0.07096 to 0.1071, P = <0.0001. **B-C**. 20-80% rise times of responses to rewards compared with S- offsets are similar for GCaMP6f. D. With dLight in the NAc, response to S- onset is slower than to S- offset or reward: Reward vs. toneOn: Mean Diff -0.2218, 95% CI of Diff. -0.3140 to -0.1296, P= 0.0005, tone On vs. tone Off: Mean Diff 0.1393, 95% CI of Diff., P = 0.045. Responses to S- onset were not monotonically decreasing for GCaMP6f so were not included in the graphs. N, depicted on the bars = mice. Error bars represent SEM. *P ≤ 0.05, **P ≤ 0.01, ***P ≤ 0.001. Source data are provided as a Source Data file. Raw data are deposited on OSF.

Supplementary Figure 9

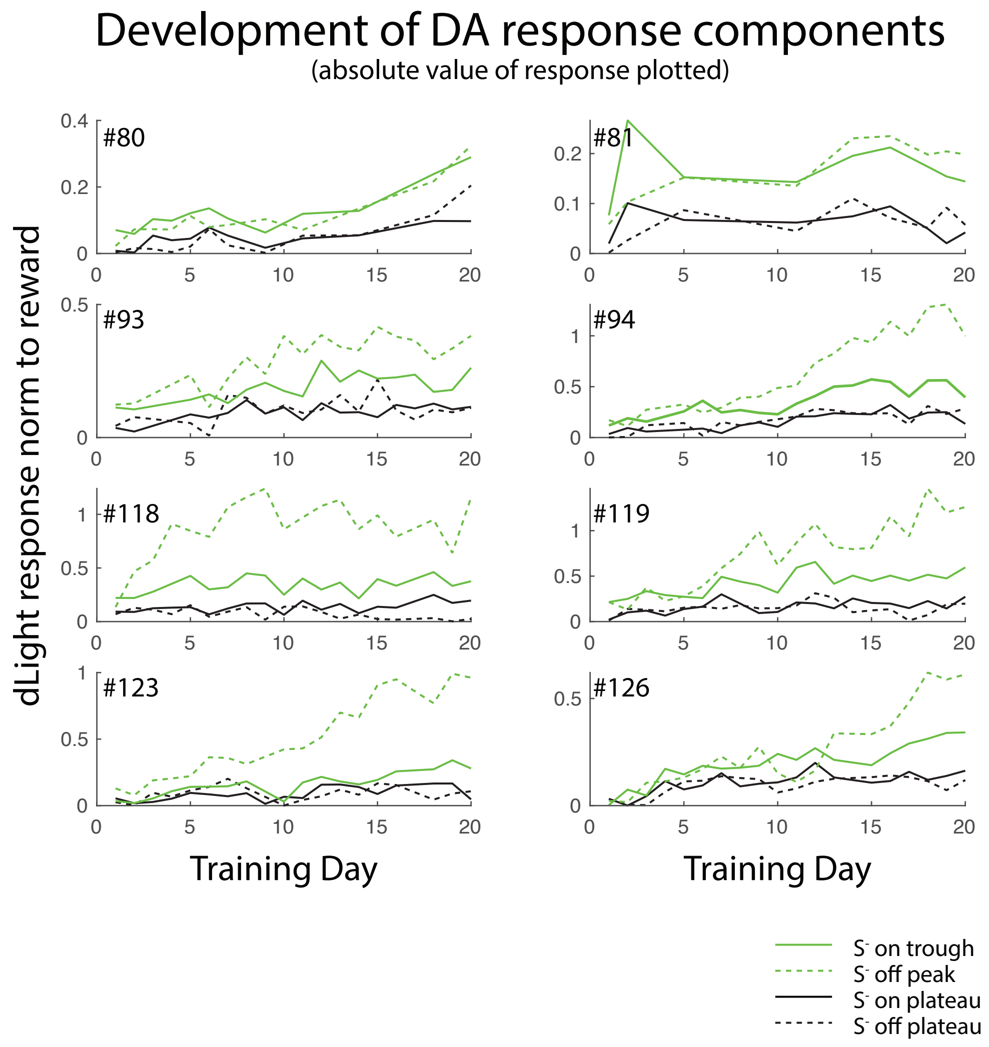

Supplementary Figure 9: Development of DA encoding components of S$^-$.
Transient responses are in green and plateau responses are in black. dLight responses are normalized to peak of average rewards response for each training day.

Supplementary Figure 10

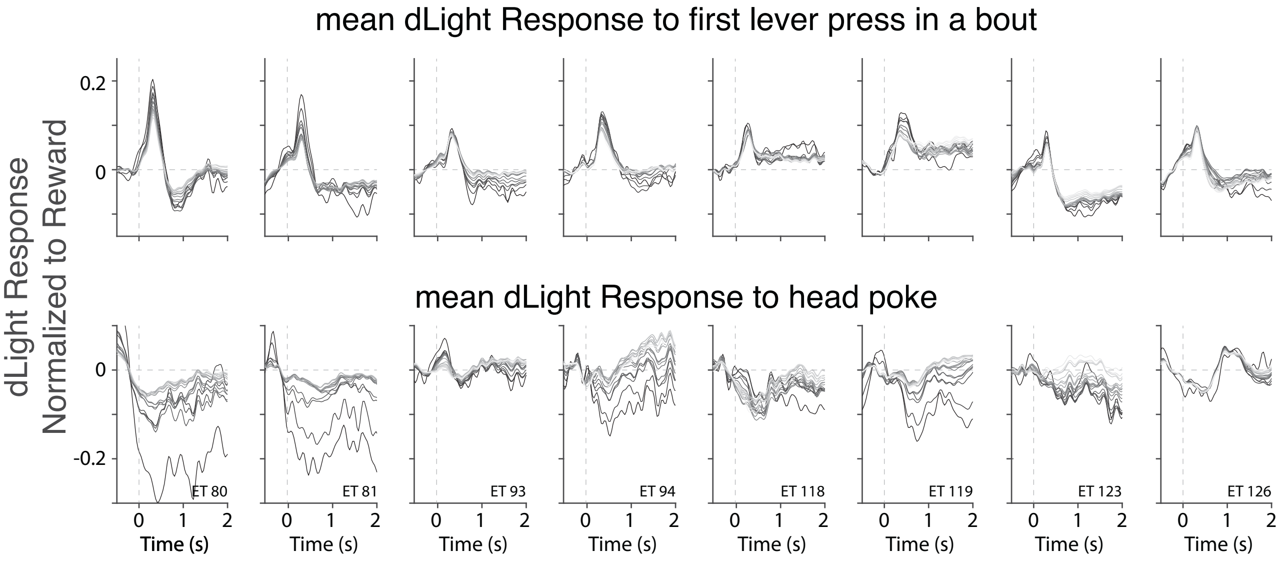

Supplementary Figure 10: Dopamine Reflects Reward Availability Before Behavioral Conditioning. (top) Mean DA response to a lever press during plateau portion of S- (25-75s of Tone S-). Each line represents average from single training day with darkest (black) lines from first training day and lightest grey line from last day. Press bouts were separated by at least 2s of no pressing. (bottom) Mean DA response to head poke during plateau portion of S- (25-75s of Tone S-). Each line represents average from single training day with darkest (black) lines from first training day and lightest grey line from last day.

Supplementary Figure 11 Conditioned Group

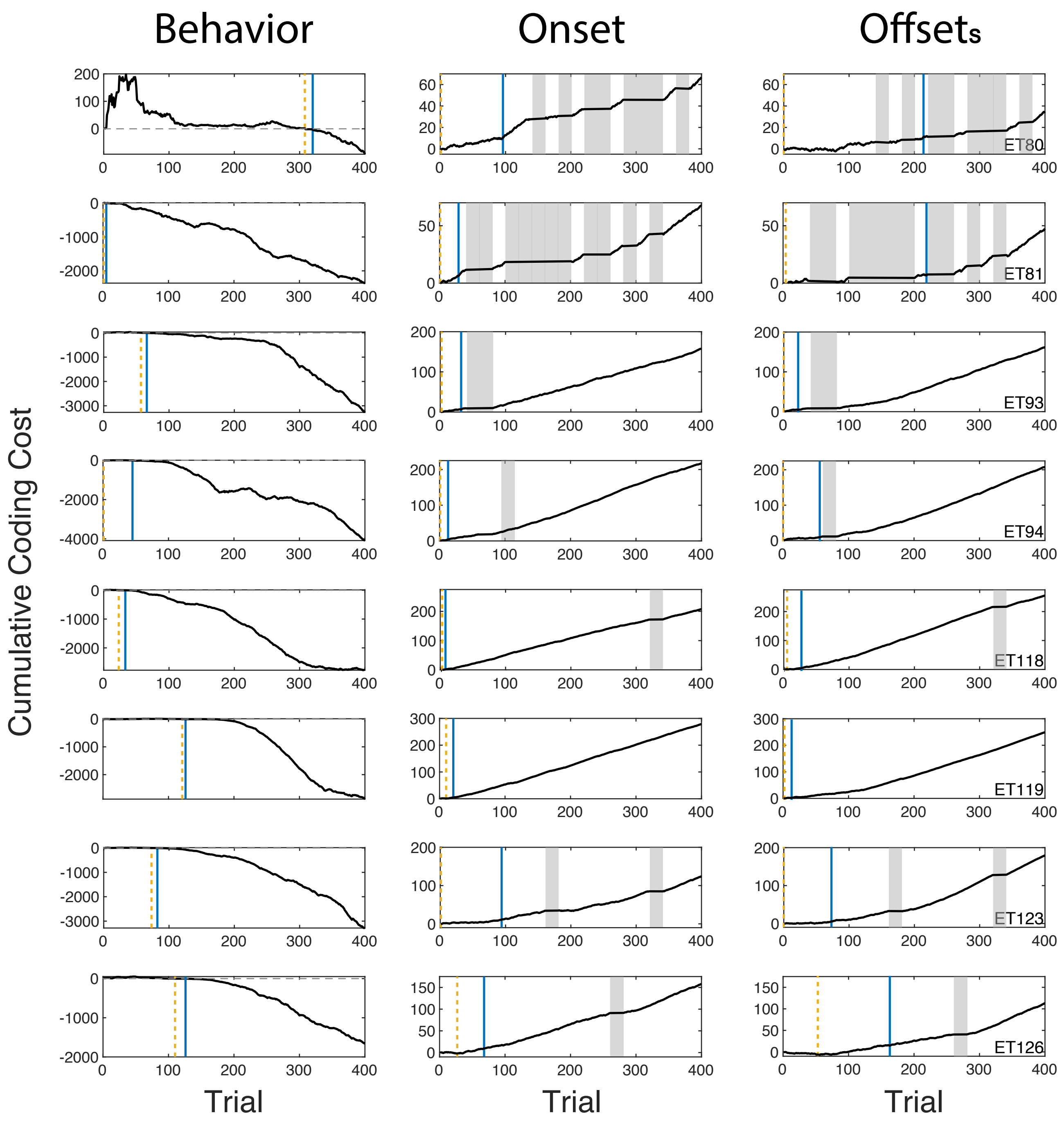

Supplementary Figure 11: Cumulative Coding Cost for each subject in conditioned group.
Cumulative coding costs as a function of trial number are presented for each subject in the conditioned group for behavior (S/ITI lever press ratio; left column), DA release (dLight) at S^- Onset (middle column) and S^- offset (right column). Grey shaded region indicates missing recording session. Vertical yellow dashed line indicates trending of difference; vertical blue line indicates signficance α = .01.

Supplementary Figure 12 Random Group

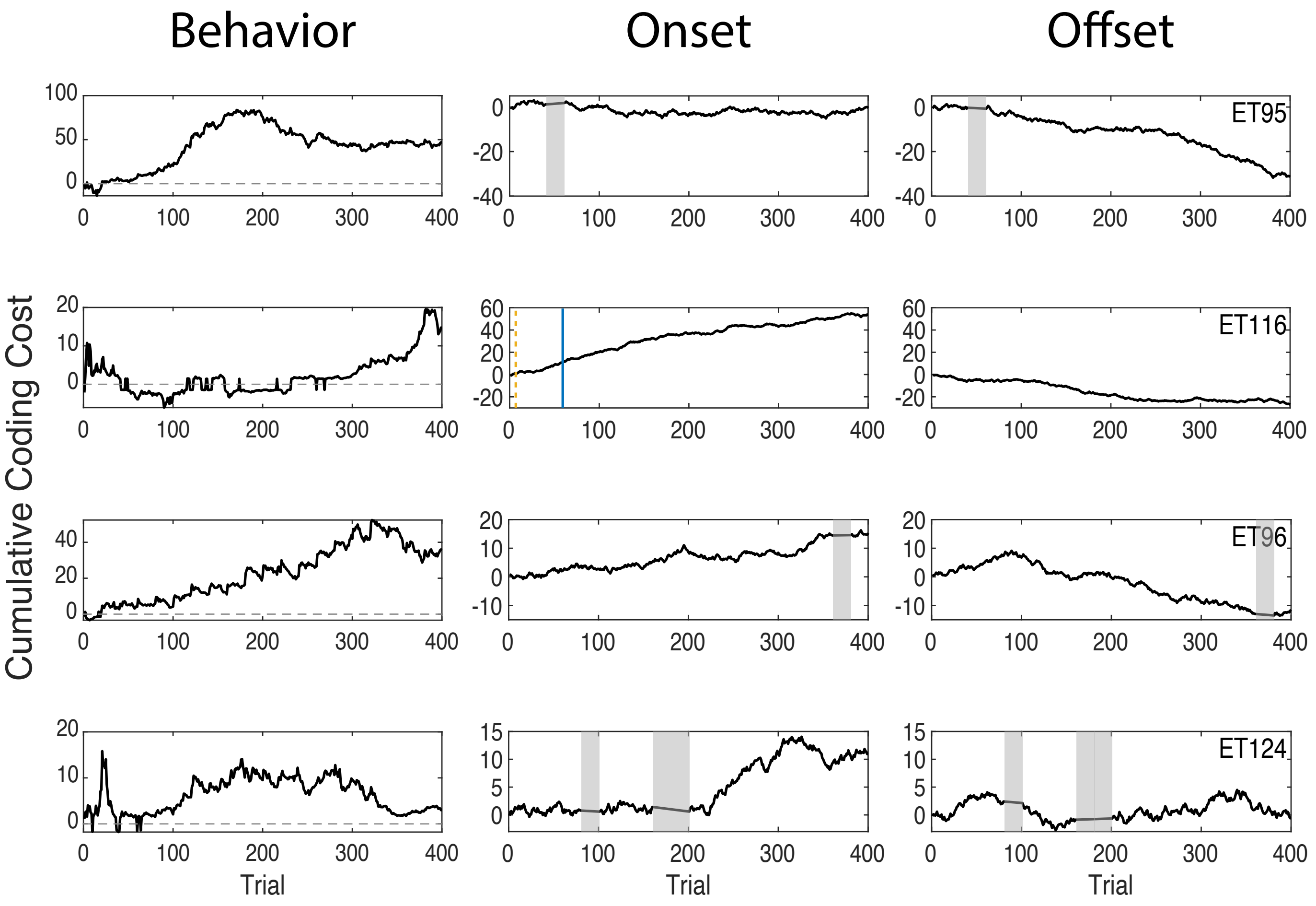

Supplementary Figure 12: Cumulative Coding Cost for each subject in random group.
Cumulative coding costs as a function of trial number are presented for each subject in the random group for behavior (S/ITI lever press ratio; left column), DA release (dLight) at S^0 Onset (middle column) and S^0 offset (right column). Grey shaded region indicates missing recording session. Vertical yellow dashed line indicates trending of difference; vertical blue line indicates signficance α = .01.

Supplementary Figure 13

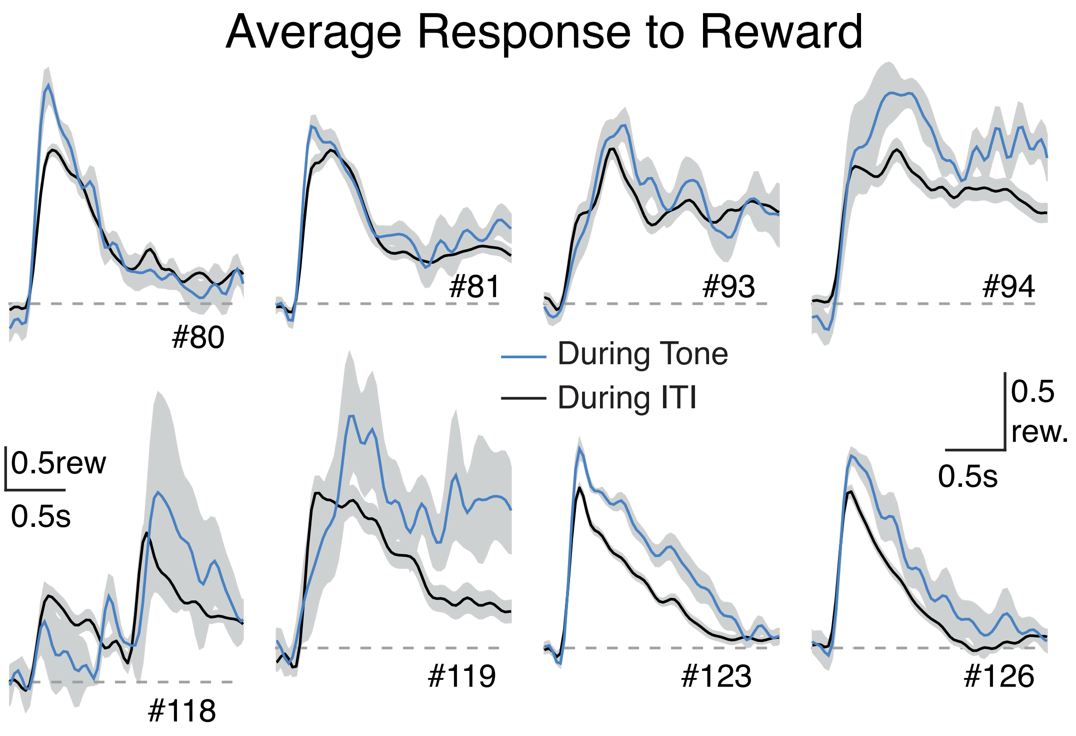

Supplementary Figure 13: Responses to rewards earned during S⁻ (blue) and ITI (black) for each of 8 subjects.

Supplementary Figure 14

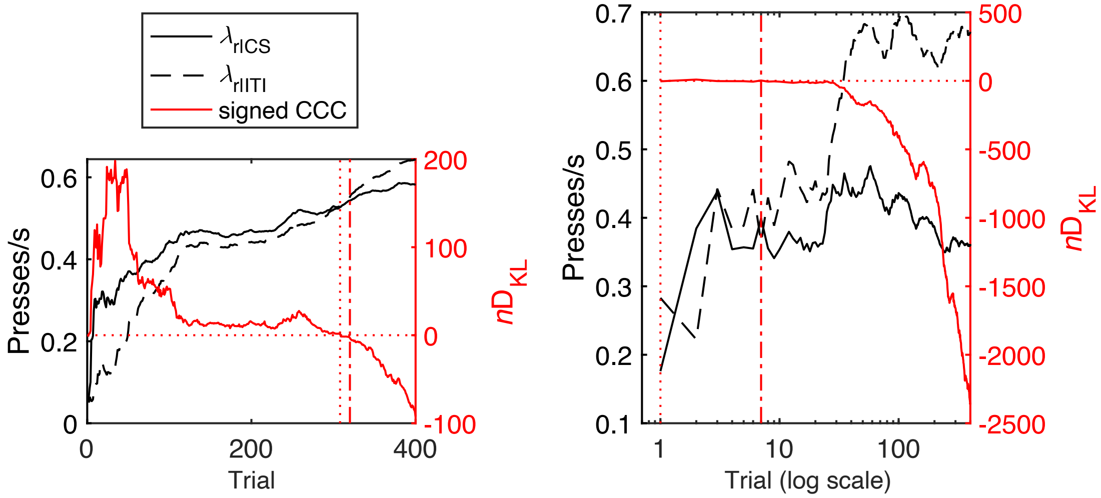

Supplementary Figure 14: Estimated lever pressing rates during the CSs, λ_(r|CS) and λ_(r|ITI) (in black, plotted against the left axes) and the signed cumulative coding cost (CCC, in red, plotted against the right axes) as a function of the training trial for two subjects. One subject was very slow to develop a conditioned response (left plot); the other developed the response very quickly (right axis). The red vertical dash-dot lines indicated the trial at which the signed cumulative coding cost became permanently less than the evidentiary criterion (a criterion that corresponds to an alpha of .01). The red vertical dot-dot line indicates the trial after which the cumulative coding cost had negative sign. This may be taken as an estimate of the trial at which the conditioned response first appeared—in contrast to the trial at which the evidence for it became strong. The x axis of the plot on the right is logged in order to reveal the very early appearance of the conditioned response. If one accepts this, then the conditioned response appeared after the first trial in the subject on the right and the evidence for it reached the .01 level of significance on the 7th trial; whereas the corresponding trials for the subject on the right are 308 (first appearance) and 320 (strong evidence). When the absolute value of the CCC is >3.32, the evidence for a divergence in the two rates is strong. Because the evidence eventually becomes overwhelming, this criterion is met when the signed CCC appears to be only slight below 0 in these plots.

Supplementary Figure 15

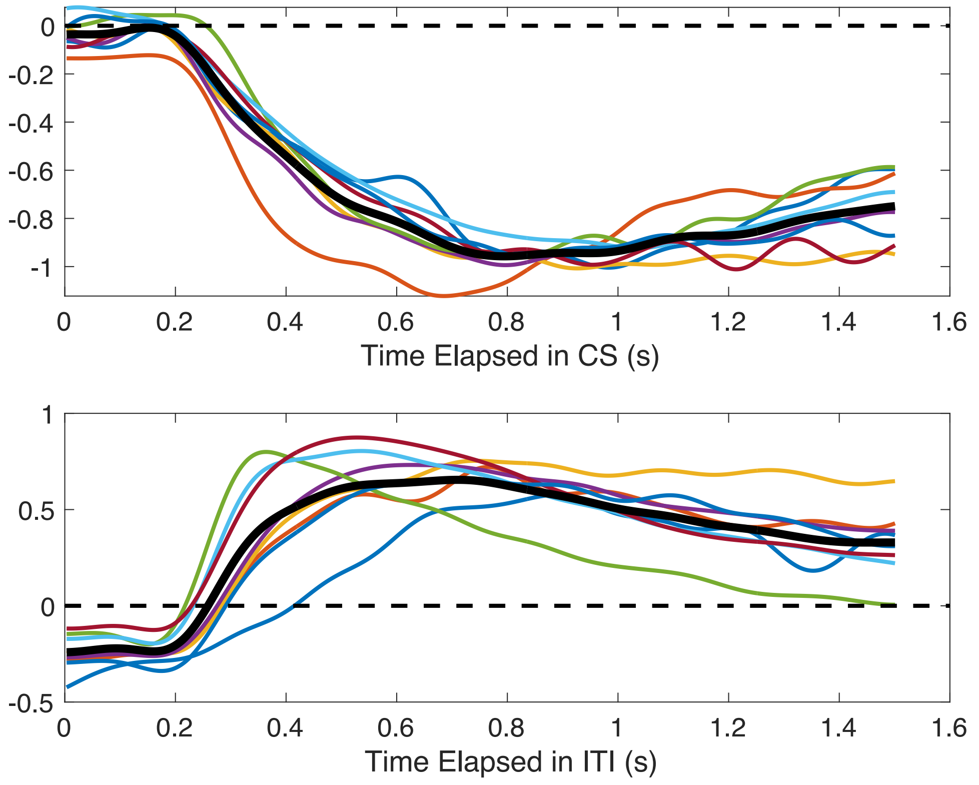

Supplementary Figure 15: The templates used to measure the extent to which the CS onset spike (top plot) and the CS offset spike (bottom plot) were present in the photometric signal on any given trial. The 8 colored curves are for the 8 subjects in the negative contingency condition. They are the average over the last 200 trials. The heavy black curves are the averages of these 8 subject-specific templates. To measure the extent to which a spike was present in the signal from a subject in the negative contingency condition, we correlated the template for that subject with the corresponding signal segment on each trial. To measure the extent to which it was present in a 0 contingency subject, we used the average for the 8 negative contingency subjects (the heavy black curve). Using the across subject average template on subjects in the negative contingency condition gave results very similar to those obtained using the subject-specific template.
