# Supplementary material for: Dopamine encodes real-time reward availability and transitions between reward availability states on different timescales
Source: Nat Commun. 2022 Jul 1;13:3805. doi: 10.1038/s41467-022-31377-2 (PMC9249893; doi:10.1038/s41467-022-31377-2)
Supplement: Supplementary file 2 — Reporting Summary [file 41467_2022_31377_MOESM2_ESM.pdf]

nature research

Corresponding author(s): Eleanor Simpson
Peter Balsam

Last updated by author(s): 05/31/2022

Reporting Summary

Nature Research wishes to improve the reproducibility of the work that we publish. This form provides structure for consistency and transparency in reporting. For further information on Nature Research policies, see our Editorial Policies and the Editorial Policy Checklist.

Statistics

For all statistical analyses, confirm that the following items are present in the figure legend, table legend, main text, or Methods section.

n/a	Confirmed	
[ ]	[x]	The exact sample size (*n*) for each experimental group/condition, given as a discrete number and unit of measurement
[ ]	[x]	A statement on whether measurements were taken from distinct samples or whether the same sample was measured repeatedly
[ ]	[x]	The statistical test(s) used AND whether they are one- or two-sided<br>*Only common tests should be described solely by name; describe more complex techniques in the Methods section.*
[x]	[ ]	A description of all covariates tested
[ ]	[x]	A description of any assumptions or corrections, such as tests of normality and adjustment for multiple comparisons
[ ]	[x]	A full description of the statistical parameters including central tendency (e.g. means) or other basic estimates (e.g. regression coefficient) AND variation (e.g. standard deviation) or associated estimates of uncertainty (e.g. confidence intervals)
[ ]	[ ]	For null hypothesis testing, the test statistic (e.g. *F*, *t*, *r*) with confidence intervals, effect sizes, degrees of freedom and *P* value noted<br>*Give P values as exact values whenever suitable.*
[ ]	[ ]	For Bayesian analysis, information on the choice of priors and Markov chain Monte Carlo settings
[x]	[ ]	For hierarchical and complex designs, identification of the appropriate level for tests and full reporting of outcomes
[ ]	[x]	Estimates of effect sizes (e.g. Cohen's *d*, Pearson's *r*), indicating how they were calculated

Our web collection on statistics for biologists contains articles on many of the points above.

Software and code

Policy information about availability of computer code

Data collection	FSCV data were acquired on custom made equipment using head-mounted voltammetric amplifier (Scott Ng-Evans, University of Washington) and controlled by custom software (TarHeel CV), written in LabView (National Instruments, Austin, TX) as described in references cited. Photometric data were acquired using two systems: Doric Neuroscience Studio (Doric) or Synapse software (TDT).
Data analysis	Custom code written for Matlab (R2019a) was used to extract and analyze the FSCV and photometric data. Those codes, as well as the MedPC codes used to run the behavioral equipment are available via OSF : https://osf.io/d4fuj<br>A detailed explanation of the cumulative coding cost method is available here:<br>https://cns2020online.sched.com/event/ctYj/w3-s10-using-cumulative-coding-cost-to-analyze-and-understand-acquisition-and-extinction<br>GraphPad Prism v8 and Microsoft Excel 2019 were used for some analyses and statistical tests.

For manuscripts utilizing custom algorithms or software that are central to the research but not yet described in published literature, software must be made available to editors and reviewers. We strongly encourage code deposition in a community repository (e.g. GitHub). See the Nature Research guidelines for submitting code & software for further information.

Data

Policy information about availability of data

All manuscripts must include a data availability statement. This statement should provide the following information, where applicable:

- Accession codes, unique identifiers, or web links for publicly available datasets
- A list of figures that have associated raw data
- A description of any restrictions on data availability

Source data are provided with this paper: the data used to generate the plots presented in this manuscript are provided in the Source Data file.
All data generated in this study (Complete Behavior, FSCV, and Photommetry datasets) used to generate the source data and the traces presented in this

manuscript (but not statistically tested) are uploaded to OSF : https://osf.io/d4fuj

Field-specific reporting

Please select the one below that is the best fit for your research. If you are not sure, read the appropriate sections before making your selection.

- [x] Life sciences
- [ ] Behavioural & social sciences
- [ ] Ecological, evolutionary & environmental sciences

For a reference copy of the document with all sections, see nature.com/documents/nr-reporting-summary-flat.pdf

Life sciences study design

All studies must disclose on these points even when the disclosure is negative.

Sample size	Samples sizes were not pre-determined but we used a minimum of 4 subjects per experiment, which is comparable to published studies in the field using similar techniques. e.g. DOI: 10.1016/j.celrep.2020.107919 fig 5, DOI: 10.1523/JNEUROSCI.0271-19.2019 fig 3.
Data exclusions	Subjects were excluded if they did not have a reward response greater than 0.25nA (for FSCV experiments) or 1 deltaF1/F2 (for photometry experiments). Individual experimental days were excluded if reward response was less than 50% of previous day's reward response. For analyses across training days, any missing days were replaced with linearly interpolated values from surrounding days. The details of missing days are provided in the Source Data File.
Replication	Every experiment, with the exception of that depicted in Figure 5f-h, contained subjects from at least 2 cohorts. Results did not differ between cohorts.
Randomization	Prior to first experimental day, FSCV or photometric responses to earned rewards were recorded. Animals were placed into groups (conditioned or random) such that the mean reward response did not differ between the two groups
Blinding	Blinding was not possible at the experimental level as the experimenter could observe whether a reward was earned during an auditory cue. Data collection was automated so blinding was not necessary. As described above, individuals were excluded based on reward responses and not conditioned stimulus responses.

Reporting for specific materials, systems and methods

We require information from authors about some types of materials, experimental systems and methods used in many studies. Here, indicate whether each material, system or method listed is relevant to your study. If you are not sure if a list item applies to your research, read the appropriate section before selecting a response.

Materials & experimental systems

n/a	Involved in the study	
[ ]	[ ]	Antibodies
[x]	[ ]	Eukaryotic cell lines
[x]	[ ]	Palaeontology and archaeology
[ ]	[x]	Animals and other organisms
[x]	[ ]	Human research participants
[x]	[ ]	Clinical data
[x]	[ ]	Dual use research of concern

Methods

n/a	Involved in the study	
[x]	[ ]	ChIP-seq
[x]	[ ]	Flow cytometry
[x]	[ ]	MRI-based neuroimaging

Antibodies

Antibodies used	Anti-GFP antibody (AbCam, ab13970) @ 1:10,000, Anti- Tyrosine Hydroxylase antibody (Millipore Sigma AB152) @ 1:1,000.
Validation	The Anti -GFP antibody recognizes variants of the Green Fluorescent Protein from the jellyfish Aequorea victoria.<br>The specificity of the GFP Antibody has been demonstrated by lack of labeling in non-transfected cells :<br>https://www.abcam.com/gfp-antibody-ab13970.html<br>The specificity of the Anti-TH antibody in IHC application has been demonstrated in numerous rodent models of dopamine depletion including PMC5499677.<br>In this study, these antibodies were used to validate the location and cell specificity of GCamp expression, not protein quantification.

Animals and other organisms

Policy information about studies involving animals; ARRIVE guidelines recommended for reporting animal research

Laboratory animals	Male C57BL/6J wild type mice (000664) were purchased from Jackson Laboratories. DAT-ires-cre mice (B6.SJL-Slc6a3tm1.1(cre)Bkmn/J) were purchased from Jackson Laboratories and bred in our animal facility to produce Male and Female heterozygous mice

	for experiments. All mice were a minimum of 10weeks of age at the beginning of experiments.
Wild animals	The study did not involve wild animals
Field-collected samples	The study did not involve samples taken from the field
Ethics oversight	All animal studies were approved by the New York State Psychiatric Institute Animal Care and Use Committee and were performed in accordance with PHS Policy on Humane Care and Use of Laboratory Animals.

Note that full information on the approval of the study protocol must also be provided in the manuscript.
